# Differentiated genomic footprints suggest isolation and long-distance migration of Hmong-Mien populations

**DOI:** 10.1186/s12915-024-01828-x

**Published:** 2024-01-25

**Authors:** Guanglin He, Peixin Wang, Jing Chen, Yan Liu, Yuntao Sun, Rong Hu, Shuhan Duan, Qiuxia Sun, Renkuan Tang, Junbao Yang, Zhiyong Wang, Libing Yun, Liping Hu, Jiangwei Yan, Shengjie Nie, Lanhai Wei, Chao Liu, Mengge Wang

**Affiliations:** 1 Institute of Rare Diseases, West China Hospital of Sichuan University, Sichuan University, Chengdu, 610041 China; 2 Center for Archaeological Science, Sichuan University, Chengdu, 610000 China; 3 Faculty of Forensic Medicine, Zhongshan School of Medicine, Sun Yat-Sen University, Guangzhou, 510275 China; 4 School of Medical Information, Chongqing Medical University, Chongqing, 400331 China; 5 School of Forensic Medicine, Shanxi Medical University, Jinzhong, 030001 China; 6 School of Basic Medical Sciences, North Sichuan Medical College, Nanchong, 637000 China; 7 Institute of Forensic Medicine, West China School of Basic Science & Forensic Medicine, Sichuan University, Chengdu, 610041 China; 8 School of Sociology and Anthropology, Xiamen University, Xiamen, 361005 China; 9 Department of Forensic Medicine, College of Basic Medicine, Chongqing Medical University, Chongqing, 400331 China; 10 School of Forensic Medicine, Kunming Medical University, Kunming, 650500 China; 11 School of Ethnology and Anthropology, Inner Mongolia Normal University, Inner Mongolia, 010028 China; 12 Anti-Drug Technology Center of Guangdong Province, Guangzhou, 510230 China; 13 Guangzhou Key Laboratory of Forensic Multi-Omics for Precision Identification, School of Forensic Medicine, Southern Medical University, Guangzhou, 510515 China; 14 Research Center for Genomic Medicine, North Sichuan Medical College, Nanchong, 637100 China

**Keywords:** Genetic diversity, Demographic history, Admixture events, Hmong-Mien speaker, Differentiated genetic structure

## Abstract

**Background:**

The underrepresentation of Hmong-Mien (HM) people in Asian genomic studies has hindered our comprehensive understanding of the full landscape of their evolutionary history and complex trait architecture. South China is a multi-ethnic region and indigenously settled by ethnolinguistically diverse HM, Austroasiatic (AA), Tai-Kadai (TK), Austronesian (AN), and Sino-Tibetan (ST) people, which is regarded as East Asia’s initial cradle of biodiversity. However, previous fragmented genetic studies have only presented a fraction of the landscape of genetic diversity in this region, especially the lack of haplotype-based genomic resources. The deep characterization of demographic history and natural-selection-relevant genetic architecture of HM people was necessary.

**Results:**

We reported one HM-specific genomic resource and comprehensively explored the fine-scale genetic structure and adaptative features inferred from the genome-wide SNP data of 440 HM individuals from 33 ethnolinguistic populations, including previously unreported She. We identified solid genetic differentiation between HM people and Han Chinese at 7.64‒15.86 years ago (kya) and split events between southern Chinese inland (Miao/Yao) and coastal (She) HM people in the middle Bronze Age period and the latter obtained more gene flow from Ancient Northern East Asians. Multiple admixture models further confirmed that extensive gene flow from surrounding ST, TK, and AN people entangled in forming the gene pool of Chinese coastal HM people. Genetic findings of isolated shared unique ancestral components based on the sharing alleles and haplotypes deconstructed that HM people from the Yungui Plateau carried the breadth of previously unknown genomic diversity. We identified a direct and recent genetic connection between Chinese inland and Southeast Asian HM people as they shared the most extended identity-by-descent fragments, supporting the long-distance migration hypothesis. Uniparental phylogenetic topology and network-based phylogenetic relationship reconstruction found ancient uniparental founding lineages in southwestern HM people. Finally, the population-specific biological adaptation study identified the shared and differentiated natural selection signatures among inland and coastal HM people associated with physical features and immune functions. The allele frequency spectrum of cancer susceptibility alleles and pharmacogenomic genes showed significant differences between HM and northern Chinese people.

**Conclusions:**

Our extensive genetic evidence combined with the historical documents supported the view that ancient HM people originated from the Yungui regions associated with ancient “Three-Miao tribes” descended from the ancient Daxi-Qujialing-Shijiahe people. Then, some have recently migrated rapidly to Southeast Asia, and some have migrated eastward and mixed respectively with Southeast Asian indigenes, Liangzhu-related coastal ancient populations, and incoming southward ST people. Generally, complex population migration, admixture, and adaptation history contributed to the complicated patterns of population structure of geographically diverse HM people.

**Supplementary Information:**

The online version contains supplementary material available at 10.1186/s12915-024-01828-x.

## Background

China is rich in human biodiversity, and over six language families exist here, including Altaic (Mongolic, Tungusic, and Turkic), Sino-Tibetan (Sinitic and Tibeto-Burman (TB)), Hmong-Mien (HM), Tai-Kadai (TK), Austronesian (AN), and Austroasiatic (AA). The genetic patterns of modern Chinese populations revealed the population stratification among ethnolinguistically different people, which was strongly correlated with geography, culture, and language families [1–3]. Recent genetic cohorts from the China Metabolic Analytics Project (ChinaMAP) [4] and NyuWa genome resources [5] have provided crucial genetic variation data from geographically different Chinese populations and offered new insights focused on population structure and the medical relevance of Chinese people. We also noticed that all these genetic studies in China mainly included Han Chinese as their major studied subjects, which would introduce the Han bias in Chinese population genetic studies and influence the health inequality of genomic benefit in the Genome-drived precision medicine era [4–6]. China had two independent agriculture innovation centers in the Yellow River Basin (YRB, millet agriculture) and the Yangtze River Basin (YZRB, rice agriculture). The abundant civilization history of social organization development and technological innovation in the middle Holocene epoch facilitated the formation of the ancient Yangshao tribe and the Dawenkou tribe in North China, the Sanmiao tribe, and the Liangzhu society in South China. Recent ancient DNA has identified the genetic differentiation between Ancient Northern East Asian (ANEA) and Ancient Southern East Asian (ASEA) since the early Neolithic period, and then they experienced extensive population admixture events along different geographical corridors [7, 8]. The patterns of evolutionary history observed in East Asia differed from those in Europe and Oceania, which had undergone large-scale population admixture and replacement processes [9]. Ancient human gene flow events outside East Asia have limited influence on the genetic backgrounds of East Asians [10]. However, ancient DNA from spatiotemporally diverse East Asians has identified regional-restricted ancient founding lineages and contributed to the reconstruction of subsequent extensive population migration and admixture events [7, 11–13]. The significant bidirectional gene flow between the YZRB rice farmers and YRB millet farmers has significantly reshaped the patterns of genetic affinity and allele frequency spectrum (AFS) among ethnolinguistically diverse East Asians [7, 13]. Population substructures were also observed in modern East Asians. Our previous genetic studies identified genetic substructures in the Amur River, Tibetan Plateau, inland and coastal South China associated with geographical locations and linguistic affiliations [2, 8, 14–16]. However, these efforts have only provided primary foundational knowledge to dissect the mystery of genetically different Chinese populations’ evolutionary and adaptive history. The fine-scale genetic structure of ethnolinguistically different Chinese populations and the patterns of genetic relationship and admixture between some Chinese populations should be further explored, especially for some ethnolinguistically underrepresented groups in South China.

HM-speaking populations include those who speak Hmongic (Miao, She, and Hmong) and Mienic (Yao and Dao) languages in mountainous areas of South China, Vietnam, and Thailand [17]. The original homeland of HM people was suggested to be in Central China, associated with Neolithic Shijiahe, Qujialing, and Daxi cultures in the middle YZRB. Historical documents showed that the expansion of pro-Han Chinese or other ANEA through Central China promoted the southward of ancient HM people [18]. The complex migration and admixture history of HM people and their interaction with other southern Chinese populations (ST, TK, AN, and AA) must be further explored. Recent findings based on uniparental markers and genome-wide evidence have identified different evolutionary processes between inland TK and HM people and between coastal AN and TK people [19–21]. Wen et al. found some HM-specific maternal lineages (B, R9, N9a, and M), and ANEA dominant lineages in HM people suggested extensive ancient matrilineal interactions between ANEA and ASEA [22]. Wang et al. recently identified the extensive population admixture between HM people and Chongqing Han [23]. Interestingly, a similar pattern of the unique and differentiated genetic structure of Sichuan Miao (SCM) has been reported by Liu et al. [24]. This work identified the close genetic relationship between SCM and Vietnam HM people. Recently, whole-genome sequencing (WGS) pilot work conducted by Gao et al. has reported the evolutionary history of HM-speaking Guangxi Yao and suggested that genetic divergence between HM and TK people occurred in the early Neolithic period (~9700 YBP) and within HM people occurred about 6700 YBP. However, the comprehensive landscape of admixture and migration patterns of geographically diverse HM people and the genetic resources of another important HM-speaking She remain unreported. With a census population size of 746,385, She is widely distributed in coastal regions of Central and South China (especially Fujian and Zhejiang) and is one of the important parts of HM people. Historians proposed different hypotheses about the origins of She people, including the Wuliang Man, Dongyi and Yue people’s descendants or Nanman and Min’s descendants. However, no genetic studies have been conducted to resolve this problem.

Comprehensively representative genomic resources from ethnolinguistically diverse populations could provide a complementary genetic landscape to understand who we are, how we get here, and why we are differentiated [11, 12, 25, 26]. Human genomic projects with high geographical and ethnolinguistic coverage can also help us understand the genetic architecture of human diseases and complex physical trait architectures [4, 5, 26–28]. More and more genetic researchers and genomic projects have identified that missing diversity in human genetic studies has hindered some medical applications and comprehensive understanding of genetic background in non-European populations as the existing European bias in medical and population genetic research [29]. Genetic analyses of worldwide people in the Human Genome Diversity Project (HGDP) [30, 31], Simons Genome Diversity Project [28], and Estonian Biocentre Human Genome Diversity Panel [26] provide the basic patterns of human genetic diversity, admixture traces, and migration models. Recently, regional genomic projects of GenomeAsia 100K Project [27], NyuWa genome resource, and ChinaMAP [4, 5] also further highlighted the importance of identifying the missing genetic diversity, patterns of genetic admixture signatures, and their medical relevance in understudied human populations [27].

Although complex population admixture models and unidentified human demographic events could be constructed based on ancient and modern autosomal genomic variations [11, 15, 30], whole-genome sequences of mitochondrial DNA and Y-chromosome could provide additional evolutionary traces based on the shared or novel haplotype groups, also referred to as the haplogroups. Poznik et al. analyzed 1244 worldwide Y-chromosome sequences from the 1000 Genomes Project (1KGP) to characterize the landscape of Y-chromosome diversity of 26 worldwide populations. Karmin et al. investigated 456 geographically diverse high-coverage Y-chromosome sequences to construct the revised phylogenetic topology with the divergence time estimation of key mutation events [32, 33]. They have reported punctuated bursts and population bottlenecks associated with the cultural exchanges among worldwide populations [32, 33]. Maternal lineages among different populations could also trace the process of population evolutionary past. Recent mitochondrial studies from modern and ancient Tibetan genomes have illuminated the Neolithic expansion processes of the YRB farmers and the Paleolithic peopling of the Tibetan Plateau [34–36]. Li et al. also reported that the maternal structure of Han Chinese was stratified along three main Chinese river boundaries [37]. It is obvious that fine-scale and large-scale uniparental genetic studies should be conducted to explore the evolutionary history of the understudied Chinese populations [38, 39].

Thus, we collected genome-wide SNP data from all HM-speaking ethnic groups, including newly genotyped coastal HM-speaking She people living in Pingshui (PSS), Guanshe (GSS), and Shanyang (SYS) from Fujian province and representative HM people with whole-genome sequences, to explore their complex genetic history and biological adaptation. We conducted a comprehensive population genetic analysis based on sharing alleles, haplotypes, and paternal/maternal lineages to explore the following key questions: (1) What’s the general landscape of population structure and admixture history of HM people? (2) How about the genetic relationship between HM people and other modern and ancient East Asians? (3) How about the genetic connection and differentiation between coastal and inland HM people (mainly referred to those living in Sichuan, Chongqing, Yunnan, Guizhou, Guangxi, Hunan, and Hubei)? (4) How about the demographic history of HM people and their interaction with surrounding neighbors? Our findings presented the entire landscape of genetic variation profile, admixture process, biological adaptative history, and medical relevance based on the HM integrative genomic resources.

## Results

### General patterns of population structure of HM people in the context of modern and ancient East Asians

**Fig. 1:**
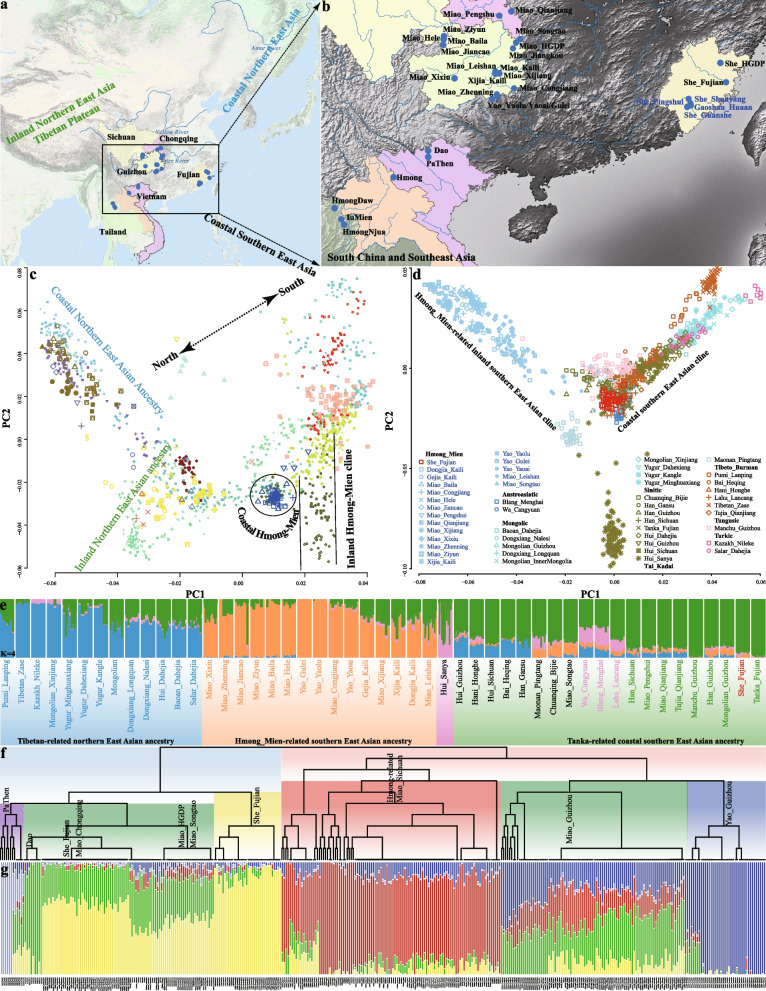
Geographical positions and population structure of Hmong-Mien (HM) people. **a**–**b** The geographical location of HM people from South China and Southeast Asia. The geographical distribution of all included HM-speaking populations was zoomed in on the regional map of South China and Southeast Asia on the right. Population names colored in blue highlight newly genotyped populations in this study, and population names colored in black denote reference HM-speaking populations. **c** Principal component analysis (PCA) showed the general patterns of the genetic background of modern and ancient East Asians based on the merged Human Origins (HO) dataset, where ancient people were projected on the PCA. Modern populations from different language families and ancient populations from different geographical regions were color-coded. The detailed legends for **c** are presented in Additional file 2: Fig. S1. **d** PCA showed the genetic patterns of Chinese populations based on the high-density Illumina dataset. **e** Model-based ADMIXTURE results obtained based on the high-density Illumina dataset with four predefined ancestral sources showed the individual or population ancestral composition. **f** Fine-scale population structure among 33 HM groups inferred from the shared haplotypes (**g**) and the ancestral composition of 33 HM groups with five predefined ancestral sources inferred based on the shared alleles. The labels in the phylogenetic tree indicated that this branch contained the majority of individuals in the labeled group. Each individual ID was marked below the ADMIXTURE results

We newly reported genome-wide SNP data from coastal HM people and merged it with our previously generated high-density SNP data of 311 individuals from 22 populations and previously published 20 HM people from HGDP (She (HGDPS) and Miao (HGDPM)) and 71 HM speakers from Vietnam and Thailand reported by Kutanan et al. and Liu et al. [40–42] and finally formed a merged HM-specific genomic dataset, including 440 individuals from 33 populations (Fig. 1a–b). We aimed to explore genetic diversity comprehensively, illuminate the evolutionary and adaptative history of geographically diverse HM people, and discover their genetic heterogeneity and connection. Representative HM-speaking individuals were whole-genome sequenced to explore their effective population size (Ne) changes and divergence times. We also merged our HM genomic resource with 929 publicly available high-coverage WGS genomes from 53 worldwide populations included in the HGDP, 317 high-coverage WGS genomes from 26 populations in the Oceanian genomic resources, or genotype data of 20,580 modern and ancient individuals included in the Human Origins (HO) and 1240K datasets from the Allen Ancient DNA Resource (AADR) [40, 43–45]. Finally, our project generated the merged high-density Illumina or Illumina_WGS dataset, the middle-density 1240K dataset, and the low-density HO dataset (Additional file 1: Table S1) that was used in different population genetic analyses. The former was mainly used for identifying natural selection signatures, and the latter two were primarily used for population admixture modeling and demographic history reconstruction.

To investigate the basic patterns of genetic admixture and the relationship between HM people and other modern and ancient populations, we conducted principal component analysis (PCA) and unsupervised model-based ADMIXTURE modeling among modern and ancient eastern Eurasians. We projected ancient people into modern population backgrounds (Additional file 2: Fig. S1). Clustering patterns based on genetic variations among eastern Eurasians showed significant genetic differentiation between ancient southern Siberians, ANEA, and ASEA/Southeast Asians (Additional file 2: Fig. S1a). We further conducted PCA focused on East and Southeast Asians to explore the fine-scale genetic relationship among regional populations (Fig. 1c; Additional file 2: Fig. S1b). We observed fine-scale population substructures associated with geographical regions and cultural features: Mongolic and Tungusic people from Northeast China clustered with Neolithic Boisman, DevilsCave, and Cis-Baikal people; TB-speaking Tibetan people clustered together and grouped closely to Nepal Chokhopani, Mebrak and Samdzong people. ASEA people were also separated into two major groups: Coastal AN people formed a genetic cline and clustered with Neolithic Fujian and Taiwan Hanben people; Inland HM-speaking Hmong, Dao, and PaThen formed a cline. However, HGDPS and HGDPM huddled closely with Han Chinese, suggesting the genetic stratification among HM people. We could confirm the identified population stratifications within regional populations from inland and coastal South China and Southeast Asia (Additional file 2: Fig. S1c). We also explored the patterns of genetic substructure among Chinese populations using the merged high-density Illumina dataset and found that the clustering patterns and ancestral components of HM people were significantly different from other reference groups (Fig. 1d–e).

The ancestry composition inferred from model-based ADMIXTURE with eight predefined ancestral sources (K = 8) (Additional file 2: Figs. S2 and S3a) showed the significant genetic differentiation between ANEA and ASEA (Additional file 2: Fig. S1a~b). We observed four ANEA-related ancestral components maximized in Lubrak, Mongolia_N_North, Tarim_EMBA1, and Jomon and four ASEA-related ancestral sources related to Htin, Mlabri, Hanben, and Yao people (Additional file 2: Fig. S3a). Ancient Gaohuahua people from Guangxi and modern HM-speaking Yao, Miao, Hmong, PaThen, and IuMien had a similar ancestral composition with the highest bright green ancestry proportion. To confirm the identified pattern of genetic diversity, we excluded reference populations with distant genetic affinity or substantial bottlenecks in our dataset. We conducted additional ADMIXTURE analyses and observed the consistent patterns of ancestry composition (Additional file 2: Fig. S1d). We found that four ancestral components were enriched in AA-speaking Htin, HM-speaking Hmong, AN-related Hanben and Ami, and northern ancestry related to Neolithic Shamanka people. When ancestral sources increased to five, inland Tibetan-related ancestry was separated from others (Additional file 2: Fig. S1d), and inland HM people showed their specific ancestral component. The genetic structure of coastal She people from Fujian first reported here showed a genetic ancestry similar to geographically close populations, not to linguistically close inland HM people (Additional file 2: Fig. S1d). Admixture scenarios and phylogenetic relationships among 33 HM groups were further explored using three clustering approaches implemented in fineSTRUCTURE, ADMIXTURE, and TreeMix (Fig. 1f–g; Additional file 2: Figs. S4~5). The resulting patterns of genetic substructures inferred by these three methods were consistent, reflecting the close genetic affinity between Chongqing Miao (CQM), Fujian She (FJS), and Songtao Miao (STM), as well as the close genetic relationship between Hmong-related people from Southeast Asia, SCM, Guizhou Miao (GZM), and Guizhou Yao (GZY). Generally, population structure inferred from PCA and ADMIXTURE suggested that complex population separation and admixture played a pivotal role in the basic landscape of modern and ancient East Asians. Geographically different HM people harbored complex and differentiated population history based on the observed patterns of genetic differentiation among geographically diverse HM populations. In the following sections, we comprehensively characterized the genetic history of previously unreported coastal HM-speaking She people and explored the global landscape of demographic history and local adaptation of all geographically diverse HM people based on state-of-the-art methods.

### Demographic history reconstruction of southeastern coastal HM people

The genome-wide variations and genetic diversity patterns of three HM-speaking She populations and one AN-speaking Gaoshan group from Fujian in southeastern China were first reported here. Results from the PCA clustering and model-based ADMIXTURE with 2 to 11 ancestral sources presented the differentiated genetic features of She people at different resolutions (Additional file 2: Fig. S6). We observed two ancestral components related to ANEA and ASEA at K = 2. The proportion of HM-specific ancestry in She people ranged from 0.902 to 0.910. ANEA ancestries related to Tibetan and Siberian people were separated from each other when K values increased from 2 to 4. When the ancestry sources were assumed to be 5~7, ASEA ancestries related to AN, HM, and AA people were separated. We found that She people were modeled with primary ancestry from Hmong-related and Tibetan-related sources and minor ancestry from AN people. Interestingly, when K values were larger than 9, we identified one blue ancestry maximized in She people.

To localize the genetic relationship between coastal She and Gaoshan people and geographically diverse modern and ancient populations, we conducted PCA analysis and found that She and Gaoshan were clustered with ASEA, which had the most intimate relationship with Fujian ancient people (Fig. 1c; Additional file 2: Fig. S1b). When we only focused on modern southern Chinese and Southeast Asians, geographically different She people clustered together and showed a close relationship with Fujian Han Chinese, but they separated from Gaoshan people and the HM cline (Additional file 2: Fig. S1c). The estimated pairwise Fst values also showed that geographically diverse She populations had the closest genetic relationship with each other and with southern Han Chinese, followed by TK people from South China (Additional file 1: Table S2). The estimated genetic drift based on the outgroup-*f*_*3*_(Eurasian, She/Gaoshan; Mbuti) further showed that each coastal She population tested, in turn, had the closest genetic relationship with geographically close She people and Han Chinese from Fujian and Guangdong provinces and also had a close genetic affinity with the late Neolithic to the Iron Age people from Henan province (Luoheguxiang and Pingliangtai, Additional file 1: Table S3).

We further performed admixture *f*_*3*_-statistics to explore the potential admixture signals of coastal HM populations. Three population tests in the form *f*_*3*_(Source1, Source2; Targets) could provide direct evidence for genetic admixture signatures (*Z*-scores < − 3). However, we found no admixture signals in geographically diverse She but identified many negative values in *f*_*3*_(Ami, Eurasian; Gaoshan), which suggested that Gaoshan people were one mixed group with one source from Ami and another associated with Han Chinese (Additional file 1: Table S4). To test which populations shared the most alleles with our surveyed She and Gaoshan people, we conducted formal *f*_*4*_-statistics in *f*_*4*_(Reference1, Reference2; She/Gaoshan, Mbuti). PSS shared more alleles with HGDPS compared with inland HM and other Asian reference populations except for Shanghai Han, Hubei Han, Chuanyun_H, Haojiatai_LN, Longtoushan_BA and Miaozigou_MN (Additional file 2: Fig. S7). Similar patterns of shared ancestry between GSS and SYS with Han Chinese/pro-Han were also identified (Additional file 2: Figs. S8~9). A different status of genetic relationship was identified in the Gaoshan people, which shared the most alleles with AN-speaking Ami and Kankanaey compared with other reference populations, followed by TK speakers and southeastern Han Chinese (Additional file 2: Fig. S10). Modern northern East Asian and ANEA shared more alleles with Fujian Gaoshan people than most AN and AA people in Southeast Asia.

Formal tests in *f*_*4*_(Han_Fujian/HGDPS, GSS; Eurasians, Mbuti) have not identified statistically significant values (Additional file 2: Fig. S11), suggesting that studied She people formed one clade with Han Chinese and HGDPS. We then tested which reference populations might be the ancestral source of the She people. We hypothesized that She people were directly derived from ANEA and then tested *f*_*4*_(ANEA, She; Eurasian reference populations, Mbuti). However, we identified many statistically negative values when we used reference populations from South China and Southeast Asia (Additional file 2: Fig. S11). Our identified signals suggested that She people obtained additional ancestry from ASEA compared to ANEA. She people similarly also obtained additional ancestry from ANEA compared with ASEA (Additional file 2: Fig. S10). We could conclude that coastal HM-speaking She harbored ancestries from ANEA and ASEA and possessed similar admixture patterns as observed in the geographically close Han Chinese. To further clarify the ancestral sources of She people, we conducted *f*_*4*_(TK/inland HM, She; Eurasians, Mbuti) and identified many negative values when we used ST people and ANEA as the reference populations (Additional file 2: Figs. S12~13), suggesting more genetic influence from ANEA to coastal HM than inland HM people. When focusing on Gaoshan people, the estimated statistically negative values of *f*_*4*_(ST/ANEA, Gaoshan; AN/TK/AA, Mbuti) suggested that Gaoshan people harbored more ancestry related to ASEA, especially AN-related sources (Additional file 2: Fig. S14). Strong shared ancestry derived from Fujian AN-speaking Gaoshan people was further confirmed by the negative values in the *f*_*4*_(Eurasians, Gaoshan; Ami/Kankanaey/Hanben, Mbuti). Results from the negative values in *f*_*4*_ (Ami/Kankanaey/Hanben, Gaoshan; ANEA, Mbuti) confirmed that Fujian Gaoshan people obtained additional genetic influence from ANEA compared with Taiwan island populations (Additional file 2: Fig. S14).

**Fig. 2:**
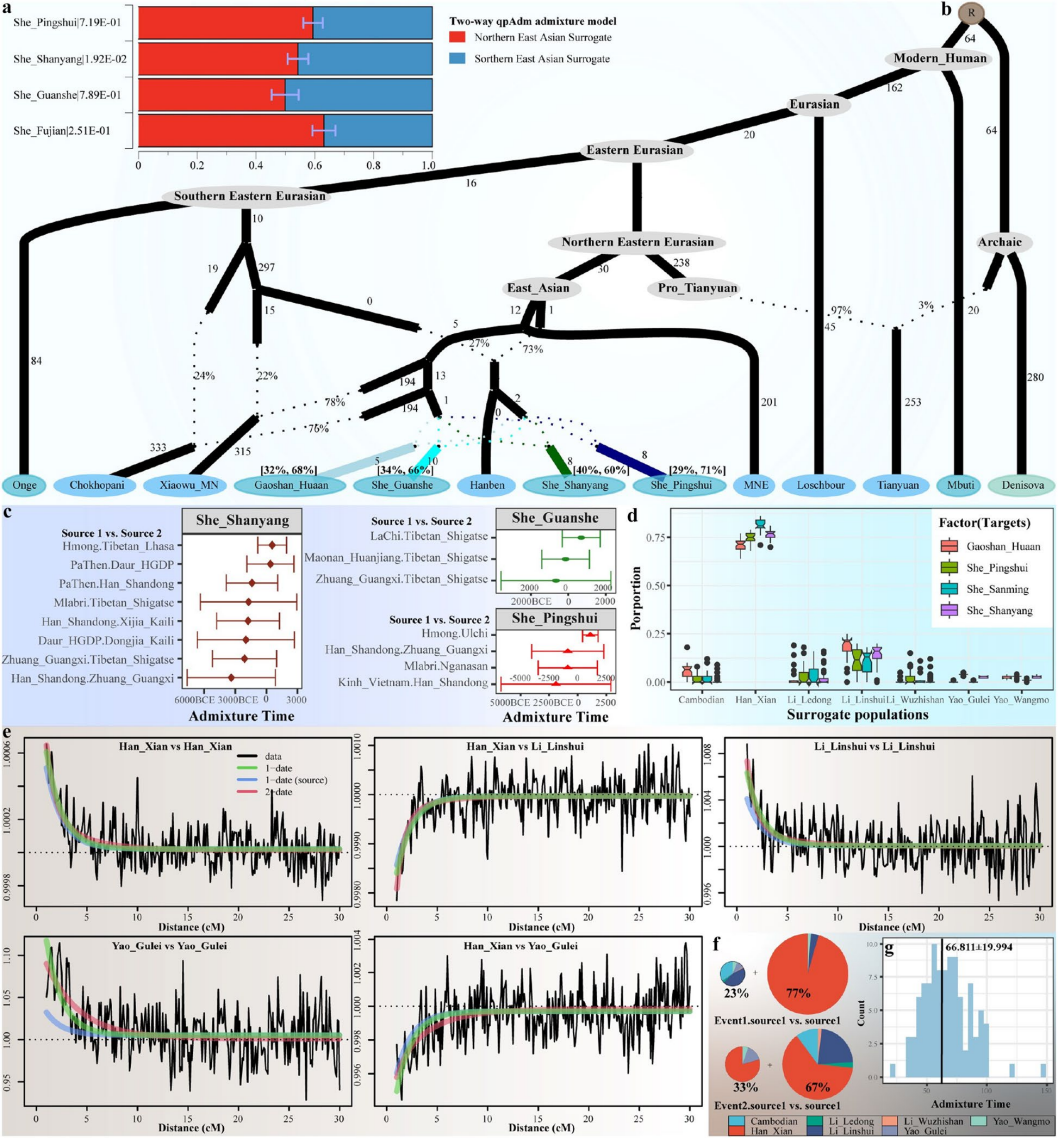
Demographic history reconstruction of the coastal Chinese HM speakers. **a** The results of the two-way qpAdm analysis showed the well-fitted models with northern and southern East Asians. The bar showed the standard error. The estimated p values were followed in the population label. **b** qpGraph model showed the evolutionary history of newly collected coastal Chinese populations and corresponding ancient East Asian lineages. The presented qpGraph-based phylogenetic topology was fitted for Gaoshan people with the best–worst Z-score of − 1.992. We manually added the admixture events of She people to this model. The *Z*-scores of the GSS people are − 1.878, 1.873 for PSS, and − 2.258 for SYS. Branch lengths were labeled in units of* f*_*2*_ genetic drift distance times 1000. The dotted line indicates the admixture events with admixture proportion. **c** Admixture times of newly genotyped coastal Chinese populations were estimated using different ancestral sources. The reference populations on the left represented the best representative source pairs. The red triangle or green circle indicated one admixture event with 95% confidence intervals, and the *Z*-score indicated the statistical deviation of magnitude from zero. **d** The boxplot showed the genetic contribution estimated via the shared haplotypes from northern and southern sources to our studied populations. **e** The estimated probability of two genomic chunks separated at certain genetic distances was inherited from the predefined different surrogate population pairs. **f** Source compositions of She people estimated with the original surrogate populations. **g** Admixture dates estimated based on the 100 bootstraps, and the black line indicated the values of mean value estimate

To explore possible demographic models for illuminating admixture models and admixture times of the targeted populations, we used different statistical methods and ancestral sources to examine the admixture sources, proportions, and times. We first used geographically close Iron Age Hanben people from Taiwan Island as the ASEA sources and ancient millet farmers from the YRB as the northern sources and then conducted qpAdm modeling to test the two-way admixture models for She and Gaoshan people. All four studied populations were modeled with primary ancestry from Hanben people and minor ancestry from Yangshao or Longshan people, which was further confirmed via qpAdm models with primary Han-related ancestry and southern indigenous Li-related ancestry (Fig. 2a). We reconstructed qpGraph-based topologies with different ANEA and ASEA lineages (Fig. 2b; Additional file 2: Figs. S15~18). We found that two-way admixture models fitted all coastal HM populations, in which Gaoshan and PSS harbored more ancestry related to Hanben people. We further dated the admixture events based on the ancestral sources from ANEA and ASEA surrogates based on the ALDER-based estimates and found that She people have shown admixture signals of northern and southern Chinese ancestry since four thousand years ago with different ancestral pairs (Fig. 2c; Additional file 1: Table S5). To further explore which populations can serve as the best ancestral surrogate populations, we conducted a Sourcefind analysis and identified that northern Han contributed most ancestry to coastal populations, followed by Li and Cambodian (Fig. 2d). Coancestry curves documented the relative probability of two chunks copied from Li and Han, confirming that these two sources contributed much ancestry to coastal HM people (Fig. 2e). The best guess of one-date-multiway from the GLOBETROTTER estimates suggested that the first admixture source, best represented by Li, and the second admixture source, represented by Han, respectively contributed 0.23 and 0.78 ancestries to the inferred genetic composition of HM people in 1937 years ago with additional gene influence from Han Chinese in multiway model (Fig. 2f–g).

### Fine-scale population differentiation between inland and coastal HM-speaking populations

We comprehensively explored the genetic structure among 349 unrelated individuals from 25 Chinese HM-speaking populations based on the merged Illumina dataset to reconstruct the fine-scale population demographic history of HM people. The constructed phylogenetic topology among 203 male individuals revealed that O1 and O2 lineages contributed the most to HM people, regarded as HM’s two major ancestral founding lineages (Additional file 2: Fig. S19a). ANEA dominant C2a/b, D1a1a, and Q1a2a lineages related to Mongolian or Tibetan people [36, 46] were also identified in HM people, suggesting that the consistent southward gene flow influenced the gene pool of our studied populations. We also constructed the phylogenic relationship using the high-density Y-chromosome SNPs to explore the pattern of population divergence and expansion (Additional file 2: Fig. S19b). Phylogenetic topology showed extensive genetic interaction among inland HM-speaking Miao and Yao, who were extensively assigned to different paternal lineages. We found that two significant lineages of O2a2a1a2a1a1 and O1a1a1a1a1a1 experienced recent expansions among HM people. O2a2a1a2a1a1 sublineages were mainly observed in Yao and geographically close inland Miao people, and O1a1a1a1a1a1 sublineages were mainly identified in She people. The different founding lineages observed among inland and coastal HM people supported the observed genetic differentiation in the PCA patterns. Although the patrilocality of East Asians was supported by previous cultural and genetic evidence, we also explored the haplogroup frequency spectrum (HFS) of maternal lineages, matrilineal phylogenetic topology, and network-based phylogenetic relationship to explore the maternal genetic structure of HM people. We observed that F, B, A, M, and N lineages contributed to the maternal gene pool of HM people and identified star-like expansions in some lineages (D4, B5, and M7), supporting the recent population expansions of ancestral populations carrying these lineages (Additional file 2: Fig. S19c). We found that B5a1c1a, F1a1c3, D4e1a, and M7b1a1 were the founding lineages of Chinese HM people based on the constructed phylogenetic topology of defined terminal lineages (Additional file 2: Fig. S19d). The mosaic distribution patterns of one targeted lineage showed frequent maternal movement among geographically diverse HM people (Additional file 2: Fig. S19d).

**Fig. 3:**
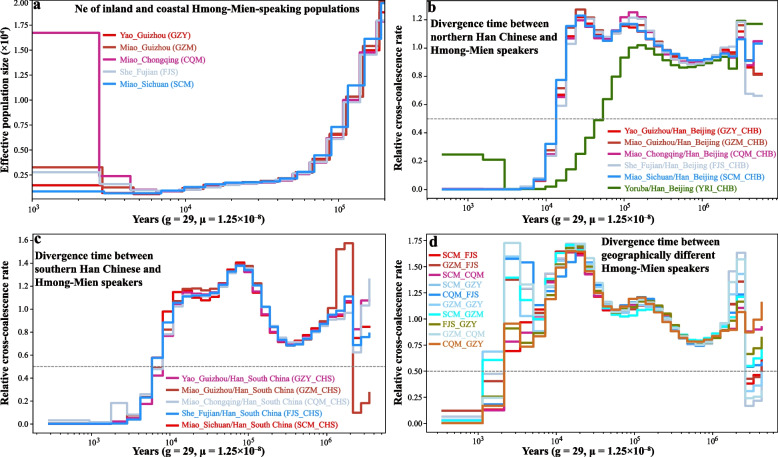
The estimated effective population size and population split times. **a** The effective population sizes of geographically different HM-speaking populations inferred using MSMC2. As shown in Additional file 2: Fig. S20, the Yoruba genome was added as a benchmark. **b** The split times between geographically diverse HM people and northern Han Chinese. The divergence time between northern Han Chinese and Yoruba was added as a benchmark. **c** MSMC2 cross-population results for pairs of geographically diverse HM people and southern Han Chinese. **d** The split times among geographically diverse HM people. GZY: Guizhou Yao, FJS: Fujian She, GZM: Guizhou Miao, CQM: Chongqing Miao, SCM: Sichuan Miao, CHB: Han Chinese in Beijing, China and CHS: Han Chinese in South China

We then estimated the effective population sizes of geographically diverse HM people and inferred the population split times among these HM-speaking populations or between targeted HM people and Han Chinese (Fig. 3; Additional file 2: Fig. S20). We added the Yoruba genome as a benchmark to confirm that our MSMC results were reliable. The effective population size of Yoruba over time and the split time between Yoruba and Han Chinese estimated in this study were similar to those revealed in a previous study [40]. We observed that CQM and FJS experienced population declines around 9000 years ago, while GZM, SCM, and GZY experienced population declines around 6700 years ago, with these HM-speaking populations subsequently experiencing population expansion (Fig. 3a; Additional file 2: Fig. S20). The effective population size of these groups stabilized around 2700‒3000 years ago, and CQM had a larger estimated population size within 100 generations relative to other HM people (Fig. 3a). The estimates of the midpoint of the relative cross coalescence rate (rCCR) showed a pre-Neolithic divergence between Han Chinese and HM people and Bronze Age separation within geographically different HM-speaking Miao, Yao and She. The split times between northern Han Chinese and HM people occurred around 15.23‒15.86 kya (Fig. 3b); between southern Han Chinese and HM people was approximately 7.64‒9.35 kya (Fig. 3c); Chinese HM-speaking populations began to separate at ~3.13 kya (SCM_FJS), and GZM and GZY finally separated at ~1.90 kya (Fig. 3d).

**Fig. 4:**
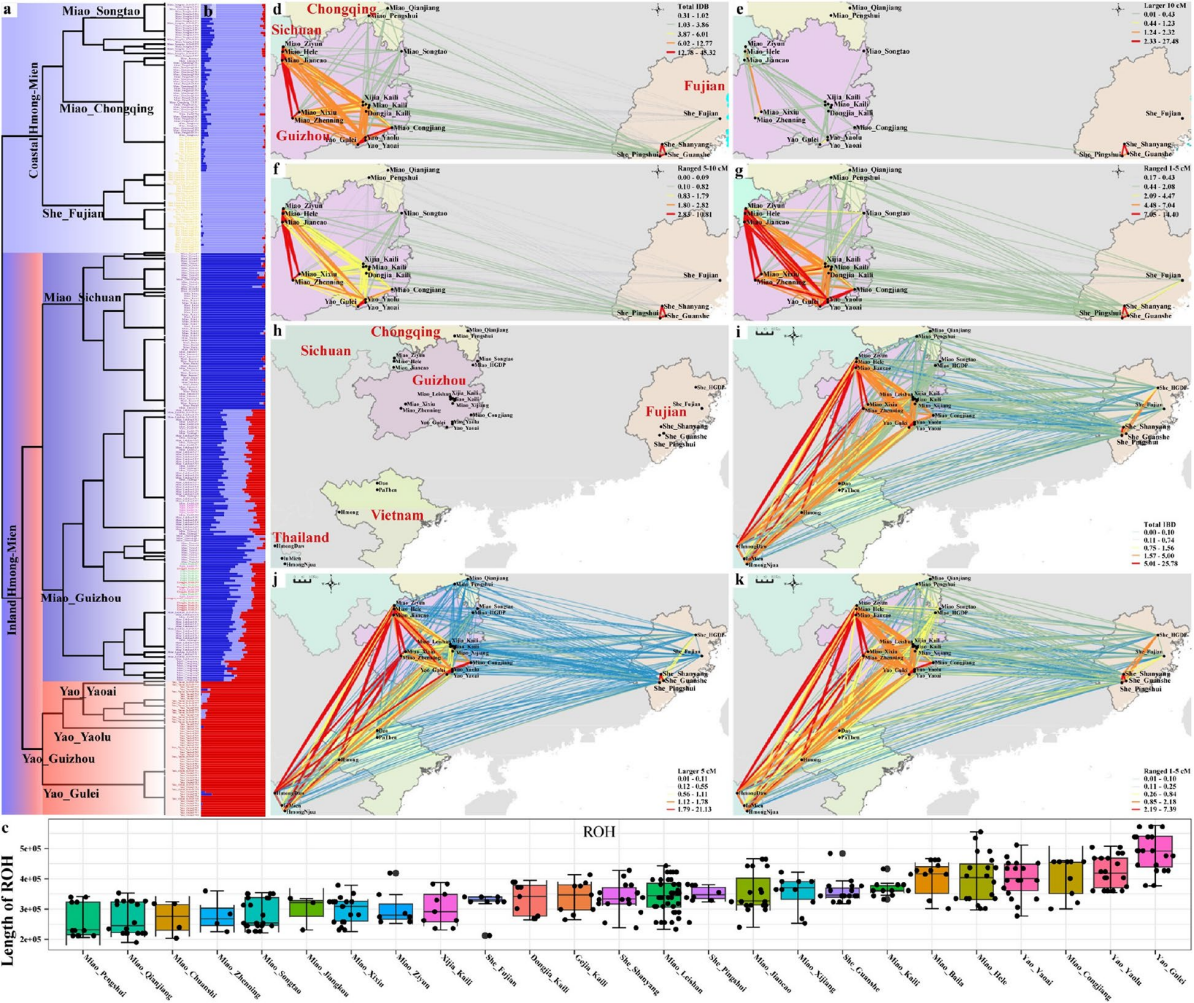
Genetic connection and affinity among HM Speakers. **a** The fineSTRUCTURE-based dendrogram showed the phylogenetic relationship among 25 HM-speaking populations in China. **b** Model-based ADMIXTURE with three predefined ancestral sources showed a consistent clustering pattern with fineSTRUCTURE results. The lowest cross-validation error value was observed when K = 3. The dusty-blue ancestral component was maximized in the FJS people, the dark-blue ancestral component was maximized in the SCM people, and the red ancestral component was enriched in the GZY people. **c** The box plot showed the results of runs of homozygosity of Chinese HM people. **d**–**g** The genetic connection among Chinese Miao, Yao and She people showed their interaction status based on the high-density Illumina dataset. **h**–**k** Shared IBD among all included HM people was estimated using the low-density merged HO dataset. **h** The dot and label showed the geographical position of HM people. The color of the lines indicates the summed IBD length, and the redder and thicker the lines, the longer the total length of the shared IBD

To explore the fine-scale population structure among 25 Chinese HM-speaking populations via the sharing alleles and haplotypes, we phased the 349 HM genomes and painted them as mosaic patterns using ChromoPainter. We then explored the genetic similarities and differences using fineSTRUCTURE based on the sharing haplotype chunks (Fig. 4a). The reconstructed dendrogram separated all Chinese HM people into two major clades and several subclades. The left clade included three subclades consisting of SCM, GZM, and GZY people. Interestingly, we found that GZY separated from the other two Miao subclades first, and then GZM separated from the SCM subclade. The top clade included CQM, STM from Guizhou, and FJS. Previous studies based on genome-wide SNP data revealed that the observed genetic diversity of CQM and STM showed similar patterns to those of geographically close Han Chinese [23, 47]. The identified genetic affinity between CQM, STM, and coastal HM people showed that these populations received significant genetic influence from Han Chinese. We further conducted an ADMIXTURE analysis to dissect the ancestral composition based on the sharing alleles (Fig. 4b). Population substructures were also identified between geographically different HM people with the best-fitted models of three ancestral sources, suggesting the differentiated evolutionary processes within HM people. Coastal HM and CQM people had the maximum light-blue ancestral component, SCM possessed the highest deep-blue ancestral component, and the red ancestral component dominated GZY people. GZM people harbored the above three ancestral components. The reconstructed identity-by-descent (IBD)-based phylogenetic topology was strongly correlated with the population stratification, as observed in the model-based ADMIXTURE results (Fig. 4a–b). The observed pattern of genetic structure among Chinese populations suggested that population isolation and admixture contributed to the formation of the gene pool of HM people. To explore whether other possible population evolutionary forces contributed to the complex pattern of genetic diversity, we estimated the runs of homozygosity (ROH) among these Miao, Yao, and She populations. We found the longest ROH indexes in Yao people than that in other populations (Fig. 4c). Miao people have recently mixed with Han Chinese and tended to have the shortest ROH values, consistent with previously evidenced admixture processes via admixture model reconstruction [23]. Our results suggested that population separation, admixture, and interbreeding contributed to the genetic differentiation among geographically different HM people.

### The direct genetic connection between Chinese inland HM and Southeast Asian HM people suggests the recent long-distance migration

Previous work has suggested that genomic variations could be used to trace the complex demographic history and recent population migration events that have resulted in the complex genetic admixture of previously geographically or culturally isolated populations, such as the Bantu expansion, the expansion of the Mongol Empire and Austronesian dispersal to Oceania [9, 43, 48]. ADMIXTURE and PCA results showed a close genetic relationship between Chinese inland HM and Southeast Asian HM people, which was also confirmed via the genetic distances (Fst and outgroup-*f*_*3*_ values, Additional file 1: Tables S2~3). Ancient Gaohuahua people from Guangxi also had a solid genetic affinity with HM people from the Yungui Plateau and Southeast Asia. To further explore genetic connections between geographically diverse HM-speaking populations based on the sharing haplotypes and visualize the correlation of their affinities with geographical position, we explored the IBD sharing between Chinese HM people based on the high-density Illumina dataset and between all HM-speaking populations from South China and Mainland Southeast Asia based on the merged middle-density 1240K dataset (Fig. 4). The estimated IBD sharing in different categories showed the extent of the population interaction in different periods. We identified extensive population interactions among Chinese HM people; we only identified an ancient connection between the coastal and inland HM people (Fig. 4g). Focused on all included HM people, we identified the recent genetic relationship between inland HM people from South China and Mainland Southeast Asia (Fig. 4h–k), consistent with the historical documents of the long-distance migrations of HM people.

### Medical relevance

**Fig. 5:**
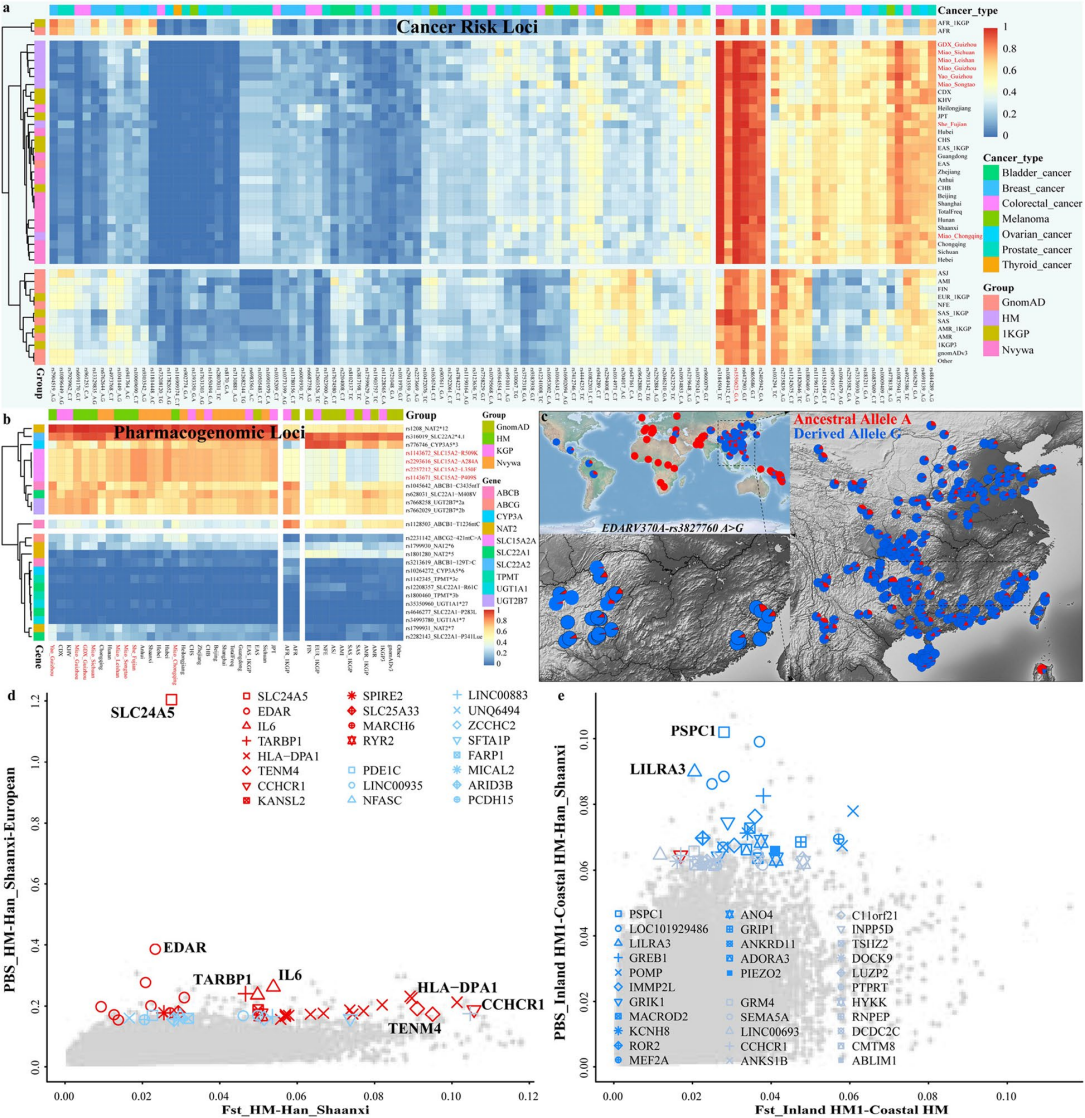
Medical relevance and natural selection signatures among HM-speaking populations. **a** Allele frequency spectrum (AFS) of 106 previously reported cancer susceptibility variants among HM people and worldwide reference populations from the NyuWa, GnomAD, and 1KGP. **b** The AFS of 25 previously reported pharmacogenomic loci in our dataset and reference groups. GDX_Guizhou included Dongjia, Gejia, and Xijia people from Kaili, Guizhou. Miao_Sichuan included Miao people from Baila, Hele, and Jiancao of Sichuan. Miao_Guizhou included Miao people from Congjiang, Xijiang, Jiangkou, Kaili, Xixiu, Zhenning, and Ziyun of Guizhou. Miao_Chongqing included Miao people from Pengshui and Qianjiang of Chongqing. Population labels in Fig. 5a–b represented only by province names represent Han Chinese from the corresponding area from the Nyuwa genomic resources. **c** Allele frequency distribution of natural selection loci of *EDARV370A*-rs3827760 A > G among 377 worldwide populations from the 10K_CPGDP, HGDP, and Oceanian genomic resources. **d** The signatures of natural selection identified in HM people based on the population branch statistic (PBS) values in the HM-Han_Shaanxi-European trio model. The X-axis denotes the Fst values for each SNP between HM people and Shaanxi Han. **e** The signatures of natural selection identified in Inland HM1 based on the PBS values of Inland HM1-Coastal HM-Han_Shaanxi trio model. The X-axis denotes the Fst values for each SNP between Inland HM1 and Coastal HM. The top 100 candidate genes located in the coding regions were highlighted. The same symbol in Fig. 5d–e represented the same gene

Population genetic databases can provide new insights into understanding complex trait architectures. Identification of risk alleles with small or large effect sizes in the cancer susceptibility genes via genome-wide association studies significantly influenced cancer clinical utility and epidemiological studies [49]. Population-based cancer prevention and screening programs depended highly on the risk discrimination estimated from the combined effect of multiple risk SNPs or individual high-effect inherited genetic susceptibility loci. Previous studies suggested that ethnolinguistically diverse populations exhibited differences in allele frequencies of genetic variants of many common and rare diseases [27, 50]. Thus, we investigated the allele frequency spectra of 255 risk SNPs in 180 genes across 27 malignancies in our HM genomes and worldwide reference populations from the NyuWa, 1KGP, and GnomAD [5]. The genetic variants with the top allele frequencies in Chinese populations were associated with the genetic susceptibility of colorectal, breast, and prostate cancers (Fig. 5a). The estimated AFS of risk alleles showed the differentiation among different continental populations and the difference between inland HM people significantly from coastal HM people and other East Asians. The frequency of rs1550623-A located in the intron region of *CDCA7* was fixed at 1 in HM people and other East Asians (Fig. 5a), and this variant was reported to be the strongest risk allele for breast cancer in Europeans (0.8469) [51]. Other risk alleles possessed a low frequency in East Asians but a high frequency in other populations or vice versa (Fig. 5a). The similar patterns of AFS in some types of cancers also showed comparable genetic basis or pleiotropy at cancer-risk loci. In addition, interpopulation differences in drug responses were generally recognized, and drugs such as clopidogrel, warfarin, carbamazepine, and peginterferon have been confirmed to show the greatest population differences in predicted adverse drug reactions [5, 27]. Thus, we assessed the AFS of 25 known pharmacogenomic variants from the ADME (absorption, distribution, metabolism, and excretion) core genes and found that some variants showed significant allele frequency differences between HM speakers, East Asians and other intercontinental populations, such as *SLC15A2* that is associated with the absorption of β-lactam antibiotics and peptide-like drugs, suggesting the necessity for genomic testing for drug response phenotypes (Fig. 5b).

### Genetic signatures of local adaptation

**Fig. 6:**
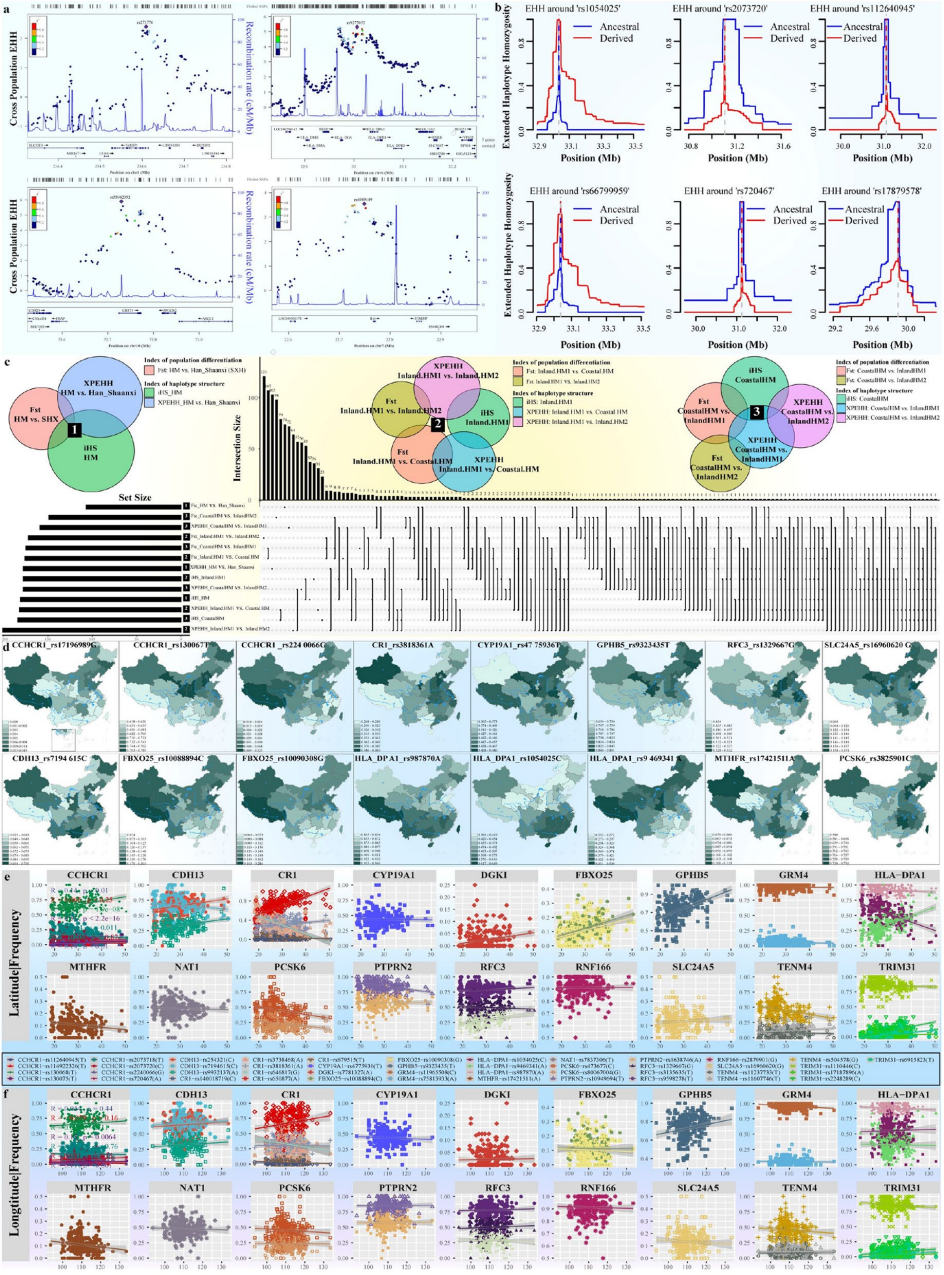
Shared and differentiated biological adaptative signatures between inland and coastal HM people inferred from different frequency or haplotype-based statistical algorithms. **a** Results of Zoom-locus showed the high-differentiated genes observed in HM-speaking populations. **b** Extended haplotype homozygosity (EHH) of core-selected SNPs. **c** Veen plots showed the shared or specific signatures of HM people inferred from different statistical designs. **d** Heat Map showed the adaptative allele frequency among 10K_CPGDP Chinese populations. **e**–**f** The correlation between minor allele frequency of selected variants and geographical coordinates (latitude and longitudes)

Genetic adaptation of different inland and coastal environments was one of the major evolutionary forces that formed the observed differentiated pattern of genetic variations. We used four different statistical methods to search for the signatures of classic sweeps among inland and coastal HM people, including population branch statistics (PBS), pairwise Fst, integrated haplotype score (iHS), and cross-population extended haplotype homozygosity (XPEHH). We conducted PBS-based genome-wide scanning using the Han_Shaanxi (SXH) and merged European populations from the HGDP as the second and outgroup reference populations to explore the HM-specific signatures of local adaptation. Several haplotype blocks with high PBS scores associated with immune functions and physical traits were identified in Chinese HM people (Fig. 5c–d). The most obvious signature of natural selection involving multiple variants inferred in the *PBS*_*HM-SXH-European*_ analysis was *EDAR*, which encodes the soluble ligand ectodysplasin A and a member of the tumor necrosis factor receptor family, regulates the changed tooth morphology, and increases scalp hair thickness [52]. Allele frequency distribution among worldwide populations included in our dataset, HGDP and 10K_CPGDP (10K Chinese People Genomic Diversity Project) suggested that East Asian and Native American people harbored a high frequency of the derived allele of *EDAR* (Fig. 5c). Spatiotemporally different ancient individuals from northeastern Asia suggested that *EDARV370A* likely increased to high frequency after the Last Glacial Maximum [11]. The following identified signals were associated with the immune system, mainly in *IL6*, *TARBP1*, *HLA-DPA1* and others (Fig. 5d), which was also confirmed via the haplotype-based analysis (Fig. 6).

*TENM4* encodes Teneurin transmembrane protein 4 and plays a role in neural development, and coiled-coil alpha-helical rod protein 1 (*CCHCR1*) that may be involved in regulating keratinocyte proliferation or differentiation were also highly differentiated variations. *SLC24A5*, which encodes an intracellular membrane protein and affects skin pigmentation [53], was another critical signal of positive selection identified in Chinese HM people with the highest PBS value but involving only one variant (Fig. 5d). We then verified the natural selection signals screened based on the PBS approach and identified additional signatures of local adaptation based on Fst, iHS, and XPEHH analyses. *HLA-DPA1*, *TENM4*, and *CCHCR1* were also identified as highly differentiated variants (HDVs) in Fst and iHS-based results (Fig. 6a–b; Additional file 1: Table S6). In addition, genes such as *CR1*, *TRIM31*, *GRM4*, and *FBXO25* were identified as HDVs based on the allele frequency differences between HM people and SXH (Additional file 1: Table S6), which showed the gradual changes of the derived allele frequency among geographically diverse Chinese populations (Fig. 6d–f). *CR1* (complement receptor 1) encodes a transmembrane glycoprotein that plays a vital role in the innate immune system. *TRIM31* (tripartite motif containing 31) encodes a protein that functions as E3 ubiquitin-protein ligase, which regulates inflammation and antiviral immune responses. The related pathway of *GRM4* is GPCR (G protein-coupled receptor) downstream signaling, which regulates neural cell proliferation and neural differentiation. *FBXO25* encodes a protein belonging to the Fbxs class and plays a role in phosphorylation-dependent ubiquitination. We also observed a robust natural selection signal at rs17421511 located in the *MTHFR*, which encodes a protein that catalyzes the conversion of 5,10-methylenetetrahydrofolate (Fig. 6a–b; Additional file 1: Table S6). The most apparent positive selection signals screened in the iHS test were *DGKI* associated with response to elevated platelet cytosolic Ca2^+^ and GPCR downstream signaling, *AHNAK* that may play a role in blood–brain barrier formation and cardiac calcium channel regulation, and *PTPRN2* related to innate immune system and PAK pathway (Additional file 1: Table S6). In addition, *CYP19A1,* which catalyzes many reactions involved in drug metabolism and lipid synthesis, and *RNF166*, involved in protein polyubiquitination, were also identified as signatures of positive selection based on the iHS scores. The signatures of positive selection identified by XPEHH scores were primarily related to the nervous system development, head development, cell–cell adhesion, regulation of cell projection organization, and actin filament-based process (Additional file 1: Table S6; Additional file 2: Fig. S21a). Generally, although most candidate signatures of natural selection were specific to one approach, most of these genes fell under the same ontology term and showed complex interactions (Additional file 2: Fig. S21b~d).

We further regrouped Chinese HM people into Inland HM1 (HM people from Sichuan and Guizhou), Inland HM2 including CQM, and Coastal HM including FJS, and then conducted a series of PBS analyses in the form *PBS*_*HM1-HM2-SXH*_ to explore the regional HM-specific selected signals. The most apparent signatures of local adaptation identified in Inland HM1 compared with the other two HM groups were *PSPC1* mediating TGF-β1 autocrine signaling and *LILRA3* encoding a member of a family of immunoreceptors (Fig. 5e; Additional file 2: Fig. S22a). *TBC1D25* related to GTPase activator activity, *WDR13* involved in a variety of cellular processes (such as signal transduction and apoptosis), *PCSK6* related to serine-type endopeptidase activity and peptidase activity, and *RFC3* related to ATP hydrolysis activity and DNA clamp loader activity were identified as candidate genes of positive selection in coastal HM people based on the *PBS*_*Coastal HM-Inland HM1-SXH*_ values (Additional file 2: Fig. S22b), and the most obvious signal of positive selection identified in Coastal HM relative to Inland HM2 was *MAGEA1*, which encodes a gene related to histone deacetylase binding (Additional file 2: Fig. S22c). We finally used allele frequency-based Fst and haplotype-based iHS and XPEHH statistical methods with the SXH as the reference population to confirm the ancient natural selection signatures and identify additional recent or ongoing signals associated with the immune system, dietary habit, and pathogen exposure. Candidate genes of natural selection involved in cell–cell adhesion (such as *CDH13*) were identified in Inland HM1 based on all five approaches (Additional file 1: Table S7; Additional file 2: Fig. S23a). In addition, signatures of positive selection related to myometrial relaxation and contraction pathways, trans-synaptic signaling, nervous system development, and cell junction organization were also identified in Inland HM1 based on any four of the five statistical methods. Statistically enriched GO (Gene Ontology)/KEGG (Kyoto Encyclopedia of Genes and Genomes) term with the largest p-value identified in Inland HM1 based on iHS scores was associated with drug ADME, such as *GPHB5* and *NAT1* (Additional file 1: Table S7; Additional file 2: Fig. S23a). The signatures of positive selection associated with cognition and circulatory system process were identified in Coastal HM based on all five approaches (Additional file 1: Table S8; Additional file 2: Fig. S23b). The statistically enriched terms related to cell–cell adhesion, dilated cardiomyopathy, modulation of chemical synaptic transmission, signaling by receptor tyrosine kinases, and Hippo signaling regulation pathways were observed in Coastal HM based on the iHS-related accumulative hypergeometric *p*-values.

## Discussion

Large-scale high-coverage WGS projects aimed at East Asians have provided insights into fine-scale population stratification, genetic diversity, dispersal, and admixture landscape [4, 5, 54, 55]. However, HM-speaking populations were significantly lacking in the early genomic projects, which are essential for better understanding the genetic diversity of South Chinese and Mainland Southeast Asians. Previous fragmented HM-related population data only reported the basic patterns of genetic structure based on the sharing alleles and were limited to presenting the entire landscape of the patterns of genetic diversity, biological adaptation, medical relevance, and population admixture models [23, 24, 41, 42]. Besides, HM people from Fujian province on China’s southern coast are under-representative in early genetic research. Thus, large-scale population genetic studies of geographically diverse HM-speaking populations are needed to fill the gap of genetic diversity in China and provide deep insights into the evolutionary history of HM people, including their origin, differentiation, and past migration.

We presented the complete landscape of population admixture, fine-scale genetic structure, and migration history at an unprecedented resolution on the HM population coverage and statistical methods. We integrated our newly generated and previously collected genomic data and formed one valuable genomic resource to illuminate the population evolutionary footprints and complex trait architecture of HM people. We extracted genome-wide SNP data from all different HM ethnicity identities, covering 440 HM people from 33 geographically diverse South China and Mainland Southeast Asian populations. We comprehensively constructed the admixture models using multiple complementary state-of-art statistical and computational methods, including traditional allele frequency-based PCA, ADMIXTURE, TreeMix, *f*-statistical tests, ALDER-based admixture time estimation and haplotype-based fine-scale population structure dissection via fineSTRUCTURE, fastGLOBETROTTER, ChromoPainter, and MSMC. We additionally explored the paternal and maternal population history via network construction and phylogenetic topology reconstruction. Generally, our results have illuminated the detailed formation process of Chinese and Southeast Asian HM people via the complex admixture model reconstruction. Fine-scale population structures of HM people identified via the sharing alleles and IBD lengths were reported at unprecedented resolution. Generally, both autosomal and uniparental genetic evidence revealed the genetic differentiation between inland and coastal Chinese HM people and demonstrated the close genetic connection between Chinese inland HM people and Mainland Southeast Asian HM people, suggesting their recent long-distance migration as documented via the historical records. Medical relevance results of cancer susceptibility genes and pharmacogenomic loci showed that genetic testing for ethnolinguistically diverse populations is essential for clinical genome medicine. Recent local adaptation to diverse environments has enabled the identification of shared and divergent natural selection signatures among geographically different HM people.

### Fine-scale population substructure of geographically diverse HM people and admixture signatures with surrounding neighbors

We used our collected genome-wide SNP data of HM people from previously understudied regions and ethnicities and merged it with all modern and ancient East Asians. The estimates of pairwise genetic distances and the patterns of genetic relationship inferred from the descriptive methods of PCA and ADMIXTURE confirmed the fine-scale population substructures in the northeast, western, inland southwestern, and southeastern Chinese coastal populations, consistent with the language belongings. Modern population structure patterns were interestingly consistent with the Holocene population structure of ancient East Asians, suggesting the strong population stability of geographically diverse ancient Eastern Eurasians. We found that HM people separated from other southern Chinese populations and formed one unique genetic cline, and 500-year-old Gaohuahua people also have a close relationship with modern HM-related cline [12]. We conducted multiple statistical methods to explore the relationship between geographically diverse HM people based on the patterns of shared alleles, haplotypes, and uniparental lineages. The genetic relationship between coastal and inland HM people revealed by PCA clustering patterns showed a differentiated affinity between the Yungui Plateau HM people and She people. The genetic relatedness revealed by ADMIXTURE, Fst, and TreeMix showed that coastal FJS people clustered closer to Han Chinese than other inland HM people. Shared genetic drift and differentiated shared ancestry inferred from *f*_*3*_/*f*_*4*_-statistics in the form of *f*_*3*_(HM, Han; Yoruba) and *f*_*4*_(inland HM, coastal HM; modern and ancient East Asians, Yoruba) confirmed the significant genetic influence of Han Chinese and TK people on coastal She people. Admixture models based on qpAdm and qpGraph also suggested more direct gene flow from ANEA participating in forming the gene pool of HM people. We also reconstructed the haplotypes of geographically different HM people, dissected their fine-scale population substructure, and differentiated admixture events. We identified two separate subbranchs according to the haplotype-based clustering patterns: one included FJS, CQM, and STM, and the other mainly included HM people from Sichuan and Guizhou. The estimated effective population sizes over time confirmed the divergent demographic history between FJS/CQM and other inland HM people, consistent with the differentiated population structure and admixture signals between inland and coastal HM people identified via allele frequency-based and other haplotype-based statistical methods. The estimated divergence times indicated that Chinese HM people began to separate about 3130 years ago, with the earliest population separation occurring between coastal She people and SCM and the latest separation occurring between GZM and GZY. Our research and an earlier study [56] suggested that proto-HM people originated from the Yungui regions and were related to the ancient “Three-Miao tribes” about 4000 years ago, and the founding paternal lineage (O2a2a1b1a1b-N5) of HM people was estimated to have diverged ~2330 years ago [21]. In general, the population divergence time between HM people computed in this study might be underestimated. We should collect more geographically diverse HM-speaking populations, especially those in Yunnan, perform high-depth WGS, and adopt advanced computational biology algorithms for modeling.

The HFS and phylogenetic topology of paternal and maternal lineages revealed the different founding lineages between coastal and inland HM people, suggesting the differentiated genetic contribution from northern and ASEA ancestral sources. The observed ANEA dominant paternal lineages (C2a/b, D1a1a, and Q1a2a) and maternal lineages (A5, C4, C7, D4, and D5) suggested that the southward gene flow from ancestral ANEA significantly influenced the gene pool of HM people. Previous studies have also found that ANEA-prevailing uniparental lineages were widely distributed in HM people [24, 47]. We found that O1a1a1a1a1a1 experienced recent expansions in She people. Previous research showed that O1a1a1 sublineages were found at high frequencies in AN and TK people [57, 58], and Taiwan Hanben individuals mostly carried O1a1 sublineages [13], indicating the substantial genetic influence of southern Chinese indigenous people on coastal HM people. Additionally, O2a2a1a2a1a1 experienced recent expansions in inland HM people. Its upstream haplogroup of O2a2a1a2-M7 occurred frequently in Daxi people from the middle reaches of the YZRB [59] and was one of the founding lineages of modern HM people [21, 24, 60, 61]. The maternal lineages B5a1c1a, F1a1c3, D4e1a, and M7b1a1, showing star-like structures and appearing frequently in targeted HM people, also exhibited relatively high frequency in ethnolinguistically diverse populations from South China and Mainland Southeast Asia [39, 60, 62]. The mosaic distribution patterns of identified uniparental lineages suggested frequent and complex gene flow between HM-related ancestors.

### Long-distance population movement among HM people and their biological adaptation

Previous modern and ancient genomic evidence has illuminated multiple southward gene flow events that influenced the gene pool of Southeast Asians [41, 42, 63, 64]. The first southward migration may be related to the migration of early YZRB rice farmers and accompanied by the spread of the AA language family. The second and third ones disseminated the AN and TK from South China to Island and Mainland Southeast Asia, respectively. The last two were associated with expanding HM people from South China and TB people from the YRB [63]. Our study aimed to explore the genetic relationship between HM people from South China and Southeast Asia based on comprehensive admixture modeling, primarily on reconstructed sharing haplotypes. PCA, ADMIXTURE, and other admixture modeling showed a close genetic relationship between Mainland Southeast Asian and Chinese inland HM people. Here, we additionally reconstructed the phylogeny based on the IBD-based genetic connection, and fineSTRUCTURE results confirmed this connection. Finally, shared patterns of different lengths of IBD showed a recent genetic interaction between HM people from South China and Thailand/Vietnam. Recent genetic studies have identified the long-distance migration in the Eurasian steppe associated with the Mongol expansion [65]. We also noted that high-depth sequencing of these samples should be conducted in the future to provide deeper insights into the admixture history and local adaptation of ethnolinguistically and geographically different HM people.

Our newly generated genomic resource was suitable for elucidating the evolutionary selective forces that contributed to the formation of our observed patterns of genetic diversity. We used four types of statistical analyses (PBS, Fst, iHS, and XPEHH) to identify the population-specific candidate signatures of natural selection associated with East Asian living conditions, infectious disease exposure, and dietary practices. East Asian-specific adaptative coding variant of rs3827760 (A > G) within *EDARV370A* showed the highest derived allele frequency in HM people. Modeling nonpathological human genetic variation in knocking mice showed that *EDARV370A* controlled the hair thickness and the number of mammary and eccrine glands [52]. Another essential human phenotypic diversity is related to skin color. Typical human pigmentation evolution genes of *OCA2*, *SLC24A5*, *SLC45A2*, and *TYRP1* were evidenced as the light-skinned selected variants in Europeans [52]. African dark-skinned related pigmentation variants were also begun to be explored. Our HM-related genetic variations also identified the most significant selection signal in *SLC24A5*, one highly penetrant genetic variant of Mendelian disorders and molecularly evidenced in the zebrafish color patterns. Recent WGS-based population analysis focused on northwestern Chinese Hui and Uyghur also identified differentiated allele frequency or haplotype homozygosity in the *SLC24A5* gene [54, 66]. Our work focused on the studied HM people and identified the highly significant selection signals of malaria infection-related *IL6* and *CR1* variants previously observed in Hainan Li [67]. Additionally, candidate genes of positive selection associated with the innate immune system, nervous system development, cell–cell adhesion, and drug metabolism were identified in Chinese HM people. We also confirmed regional HM-specific signals, such as *PSPC1*,* LILRA3*, and *CDH13*, specific to Inland HM1 relative to the other two HM groups, *TBC1D25*, *WDR13*, *PCSK6*, and *RFC3* specific to Coastal HM relative to Inland HM1, and Coastal HM-specific *MAGEA1* compared with Inland HM2, suggesting the differentiated adaptation processes of geographically diverse HM people. Still, other southern Chinese-dominant selection signatures related to malaria infection (*FREM3*) and fat metabolism-related genes (*FADS*) were not observed, suggesting that further high-depth WGS data from more ethnolinguistically diverse populations with larger sample sizes were needed in the Asian genomic projects.

## Conclusions

We generated the most extensive genomic dataset, including 440 HM people from 33 populations, to present the global pattern of genetic admixture, adaptative history, and fine-scale population genetic structure of geographically diverse HM people. We conducted population admixture modeling and demographic history reconstruction based on the shared alleles and haplotypes and supported the idea that proto-HM people originated from the Yungui regions associated with the ancient Daxi-Qujialing-Shijiahe ancestors. Fine-scale genetic structure dissection and deep demographic history reconstruction illuminated the substantial genetic differentiation between inland and coastal HM people and found that ANEA ancestry contributed more to coastal HM people than to inland ones. ROH, effective population size estimation, and other admixture signatures showed that multiple evolutionary forces, such as bottleneck and isolation, contributed to the observed genetic stratification of HM people. We found that IBD-based genetic evidence directly supported the association between long-distance population movement and admixture of HM people and the spread of the HM language family. Evidence from the adaptative history reconstruction and medical relevance analysis emphasized the shared and differentiated patterns of genetic variants or selected loci in disease susceptibility. Generally, our results provided deep insights into the formation of HM people. However, previous genetic studies based on low-density variants, small sample sizes, and local regions will miss massive population-specific genetic variants and affect the dissection of fine-scale genetic structure, complex demographic history, and local adaptation. Hence, large-scale and high-depth WGS projects covering large sample sizes and multiple geographical regions should be conducted to elaborate the evolutionary history of HM people and other understudied ethnolinguistically distinct Chinese populations. In addition, the Telomere-to-Telomere and pangenome reference genomes should be adopted for sequence alignment to detect more complex variants.

## Methods

### Human subjects, genotyping, and quality control

We collected 349 HM individuals from 25 ethnically or geographically diverse populations (Miao, Yao, and She) from Sichuan, Chongqing, Guizhou, and Fujian provinces in South China (Fig. 1a; Additional file 1: Table S1), where 38 She people (SYS: 14; PSS: 7; GSS: 17) from Fujian in coastal South China were first reported here. We also sampled four AN-speaking Gaoshan people in Fujian to explore the genetic interaction between coastal HM and AN populations. We genotyped 661,134 autosomal, 28,320 X-chromosomal, 24,047 Y-chromosomal, and 3746 mitochondrial SNPs in all HM people and Gaoshan people using the Infinium Global Screening Array (Illumina, CA, USA). We used PLINK v.1.90 [67] and King [68] to explore the close relatives within three generations. We estimated the PI_HAT values using PLINK with the “--genome” parameter. The kinship coefficients of individual pairs with PI_HAT values larger than 0.15 were further estimated using King with the “--related --ibs” parameter. We used PLINK v.1.90 [67] to filter out the variants with missing call rates exceeding 0.05 (--geno: 0.05) and remove samples with missing call rates exceeding 0.1 (--mind: 0.1). Additionally, variants with minor allele frequencies less than 0.05 (--maf 0.05) and not in Hardy–Weinberg equilibrium (--hwe 1e-6) were filtered out. The final HM-related Illumina dataset included 533,935 SNPs.

### Ethics approvement

All included individuals signed the written informed consent forms and were unrelated indigenous people in the sampling places. We also provided the necessary genetic counseling and healthy genetic reports for the sample donors if they were interested. The study protocol was approved via the medical Ethics committees at North Sichuan Medical College and West China Hospital of Sichuan University.

### Dataset arrangement and reference populations

To present a fully resolved picture of the genetic diversity of HM people, we also collected 20 HM people (10 Miaos from Hunan and 10 Shes from Fujian) from the HGDP [69] and 71 HM individuals (12 Daos, 8 IuMiens, 12 PaThens, and 39 Hmongs) from previously investigated populations from Vietnam [41] and Thailand [42] that were genotyped using the Affymetrix Human Origins array (personal communication). These HM people were merged with the above HM-related Illumina dataset to generate an HM-specific dataset, which consisted of 56,814 SNPs and included 440 HM people from 33 populations belonging to seven ethnic groups. To explore the genetic structure of HM-speaking populations in the context of modern eastern Eurasian reference populations, we first merged our HM-related Illumina dataset with published genome-wide SNP data that was genotyped using the same Illumina array to generate the high-density Illumina dataset [2, 15, 16, 23, 24, 47, 70–79]. The Illumina dataset contained 533,935 SNPs and also included two AA-speaking Blang and Wa; nine Mongolic-speaking Baoan, Dongxiang, Mongolian, and Yugur; Sinitic-speaking Han and Hui populations from Guizhou, Sichuan, Fujian, Gansu, and Hainan provinces; six TB-speaking Pumi, Bai, Hani, Lahu, Tibetan, and Tujia; one Tungusic-speaking Manchu; and two Turkic-speaking Kazakh and Salar (Fig. 1d). The high-density dataset was mainly used to perform the haplotype-based analyses and phylogenetic reconstruction of uniparental lineages. We then merged the high-density Illumina dataset with modern and ancient populations genotyped via the Affymetrix Human Origins array from the AADR [80] to form the merged low-density HO dataset, including 56,814 SNPs, which was used to explore the general patterns of population structure as this dataset included more modern and ancient reference populations. We then imputed the low-density genome-wide SNP data of modern populations in the merged HO dataset using the WBBC (Westlake BioBank for Chinese) and 1KGP haplotype reference panels [31, 81], which generated the imputed merged HO dataset covering 458,786 SNPs. The HO modern reference populations included 343 TK people from 26 populations in China and Southeast Asia, 27 Han Chinese people from 6 populations, 276 TB people from 30 Chinese and Southeast Asian populations, 224 AA people from 18 populations, 115 AN people from 13 populations, 30 Japanese and 6 Korean, 140 Mongolic people from 18 populations, and 62 Tungusic people from 62 populations (Additional file 1: Table S1) [13, 40–42, 71, 82–84]. To analyze the comprehensive admixture and interaction landscape between HM people and other ancestral source groups, we merged the high-density Illumina dataset with ancient eastern Eurasians included in the 1240K dataset to form the merged middle-density 1240K dataset, including 146,802 SNPs. Ancient eastern Eurasians were included in both the merged HO and 1240K datasets, which included 47 ancient YRB farmers from 19 populations in Shandong, Henan, Shaanxi and Qinghai [7, 13, 85]; 30 ancient people from 13 populations in Amur River Basin or West Liao River Basin [11, 85]; 23 ancient people from 9 populations in Guangxi province [12]; 54 ancient people from 9 populations in Fujian province and Taiwan island [7]; 26 ancient people from 10 populations in Japan and Korea Peninsula [10]; 33 ancient people from 7 populations on the southern fringe of the Tibetan Plateau in Nepal [86, 87]; 54 ancient people from 9 populations in southern Russia around the Baikal regions [9, 13, 25, 88–90]; 243 ancient people from 20 populations in the Mongolian Plateau [25]; 18 ancient people from 4 populations in Xinjiang [91]; and a 40,000-year-old Tianyuan individual from Beijing [92]. In addition, the Illumina dataset was also merged with the HGDP and Oceanian genomic resources to generate the merged Illumina_WGS dataset, which included 460,678 SNPs and was mainly used for identifying signals of natural selection.

### Global ancestry inference

#### Principal component analysis

We conducted PCA using the smartpca package in the EIGENSOFT v.7.2.1 [93] using all modern Chinese populations in the merged Illumina dataset or all modern and ancient eastern Eurasian populations in the merged HO dataset. When ancient people were included in the PCA analysis, we projected ancient populations into the top two coordinates extracted via modern people with the additional parameters (numoutlieriter: 0 and lsqproject: YES). To explore the fine-scale population structure, we subsequently removed modern and ancient people from southern Siberia and northern East Asia. We reran PCA based on the included reference populations from the merged HO dataset. We plot the scatter diagram using R version 3.5.2 and the in-house scripts.

#### Model-based unsupervised ADMIXTURE

We merged our data with different modern and ancient reference populations and ran model-based ADMXUTRE [94] to explore the population substructure among ethnolinguistically diverse or spatiotemporally different East Asians. Using the high-density SNPs in the Illumina dataset, we ran ADMIXTURE by merging our data with Chinese minority ethnic groups to avoid the large sample size of Han Chinese populations in the model fitness. To explore the genetic similarities and differences, we also ran ADMIXTURE using the merged low-density HO dataset. The unsupervised model in the ADMIXTURE v.1.3.0 was used here. We used PLINK v.1.90 [67] and additional parameters (--indep-pairwise 200 25 0.4 and --allow-no-sex) to remove successive variants with substantial linkage disequilibrium (LD, a squared correlation larger than 0.4) in the 200 SNP sliding windows with an SNP step of 25 SNPs. We ran the admixture models with the predefined ancestral sources (K values) ranging from 2 to 20 and used 100 bootstrap replicates (-B100) and tenfold cross-validation error (--cv = 10) to choose the best-fitted models. After pruning the linked SNPs, we used 222,526 SNPs in the Illumina dataset and 45,725 SNPs in the merged HO dataset.

#### Pairwise Fst genetic distances

To evaluate the genetic affinity among different populations in these reference panels, we used PLINK v.1.90 [51] to estimate Fst genetic distances among each population pair. Pairwise genetic distances were designed with two parameters (--within and --keep-cluster-names).

#### Inference of population admixture events

To construct the phylogenetic relationship among these ethnolinguistically diverse populations, we performed phylogenetic reconstruction using TreeMix v.1.13 [95]. PLINK v.1.90 [67] was used to evaluate the allele frequency of each population, which was used as the input file in the TreeMix-based analysis. We adopted the French population from the 1KGP as the outgroup (-root French) and ran TreeMix with the migration edges ranging from 0 to 7 and five replications for each run to explore the possible gene flow events. We used the plotting_funcs.R script to visualize each model’s phylogenetic topology and corresponding residual matrix. We used the -k flag (-k 500) to group SNPs to account for the LD. Additional parameters (-bootstrap and -global) were also used to get the best-fitted model. We also ran MEGA (Molecular Evolutionary Genetics Analysis) [96] based on the Fst genetic matrix to validate the obtained phylogenetic topology, and we obtained the consistent pattern of the major clades.

#### Runs of homozygosity

We estimated the indicator of genomic autozygosity using PLINK v.1.90 [67] based on the high-density Illumina dataset. We set the ROH containing at least 50 SNPs and a total length ≥ 500 kilobases using two parameters (--homozyg-snp 50 and --homozyg-kb 500). Two consecutive SNPs more than 100 kb apart (--homozyg-gap 100) were regarded as independent ROH. The default settings of at least one SNP per 50 kb on average (--homozyg-density 50), the scanning window contains 50 SNPs (--homozyg-window-snp 50), a scanning window hit should contain at most one heterozygous call (--homozyg-window-het 1) and the hit rate of all scanning windows containing the SNP must be at least 0.05 (--homozyg-window-threshold 0.05) were used. We further visualized the ROH distribution of each studied population statistically using R v.3.5.2 via the box plots.

#### Shared genetic drift and admixture signal estimation based on shared alleles

To measure the genetic affinity directly within HM people and among HM and other geographically close modern populations, we performed outgroup *f*_*3*_-statistics using the *qp3pop* program in ADMIXTOOLS [44]. As the merged HO dataset included the most comprehensive modern and ancient reference populations, we used *f*_*3*_(HM people, modern Eurasian; Yoruba) to explore the shared genetic affinity between HM people and modern reference populations and used *f*_*3*_(HM people, ancient Eurasian; Yoruba) to measure their genetic relationship with ancient reference populations. We also conducted the three population tests based on the merged Illumina and 1240K datasets. Similarly, we conducted admixture *f*_*3*_-statistics in the form *f*_*3*_(ancestral source1, ancestral source2; HM people) to identify the possible ancestral sources that can produce statistically significant values based on the three datasets. Here, negative *f*_*3*_ values with a Z-score lower than − 3 indicated that two ancestral sources might be the ancestral source proximities of the targeted populations and also confirmed that the studied population was an admixed population.

#### Genome-wide admixture models based on the *f*_*4*_-statistic tests

We conducted four population tests for targeted HM people based on individual and merged populations. We used qpDstat in ADMIXTOOLS [44] to conduct the *f*_*4*_(HM1, HM2; reference populations, Mbuti), *f*_*4*_(reference population1, reference population2; studied populations, Mbuti), and *f*_*4*_(reference population1, studied populations; reference population2, Mbuti). The first form was used to explore the genetic homogeneity and heterogeneity between two included HM populations. The latter two formats were used to test the differentiated genetic ancestry between our targeted and reference populations. We also used qpWave to confirm the genetic homozygosity between two HM-speaking populations and used qpAdm [44] to estimate the admixture proportion with the following outgroups: Mbuti, Russia_Ust_Ishim, Russia_Kostenki14, Papuan, Australia, Mixe, Russia_MA1_HG, Onge, Atayal, and China_Tianyuan. We next used qpGraph to test the optimal frequency-based admixture models with gene flow events among various alternative models [44].

#### Admixture time estimation based on the decay of LD

Population admixture can introduce the exponential decay of LD. We used MALDER to test the admixture LD decays and estimate the possible admixture times of HM people [97]. We used multiple modern northern and southern East Asian populations as potential ancestral sources and tested all possible source combinations. The exponential curve fitting processes added the minimum distance between two SNP bins (mindis: 0.005 in Morgan) and leave-one-chromosome-out jackknifing (jackknife: YES).

### Haplotype-based fine-scale population structure reconstruction

#### Segmented haplotype estimation

A stricter filtering strategy of missingness per SNP and missingness per individual was performed using PLINK v.1.90 [67] with two parameters (--geno 0.01 and --mind 0.01). We used the Segmented HAPlotype Estimation & Imputation Tool (SHAPEIT v2.r904) [98] to estimate haplotypes based on the high-density Illumina dataset and modern populations included in the merged HO dataset. Phased haplotypes were estimated with the following parameters to find a good starting point for the estimated haplotypes and get more parsimonious graphs: the number of burn-in iterations of 10 (--burn 10), the number of iterations of the pruning stage of 10 (--prune 10), and the number of main iterations of 30 (--main 30). We used the default settings of model parameters and HapMap phase II b37 as the genetic map in the haplotype estimates. The obtained haplotype data was used to explore the fine-scale population structure via fineSTRUCTURE, identify ancestral proximity and estimate their admixture proportion and time, and screen the natural selection signatures for local adaptation.

#### Admixture events inferred from ChromoPainter and fastGLOBETROTTER

To identify ancestral sources, date, and describe admixture events of our targeted HM people, we used ChromoPainterv2 [71] to paint the ancestral haplotype composition of our sampled HM populations. We merged our data with 929 lift-over high-coverage whole-genomes from 54 worldwide ethnolinguistically diverse populations and obtained haplotype data using SHAPEIT v2.r904 [98]. Han people from Xi’an (Han_Xi’an), Tibetan_Zase, Daur, Yakut, and Hezhen were used as the potential ANEA sources of our targets, and Yao_Gulei, Yao_Wangmo, Li_Ledong, Li_Linshui, Li_Wuzhishan, and Cambodian were used as the potential ASEA sources. We also included other Eurasian populations as the potential ancestral sources in the painting process, including Basque, French, Balochi, Brahui, Pathan, and Sindhi. All targeted HM people were used as the recipients, and geographically diverse sources were used as the donors. The estimated copying vector and painting samples showed the genome-wide haplotype sharing patterns used to run fastGLOBETROTTER based on the default parameters [99]. We also used sourcefindV2 to confirm the close genetic affinity between our targeted populations and used ancestral surrogates.

#### Painting chromosomes and fineSTRUCTURE analysis

To dissect the fine-scale population stratifications, we used ChromoPainterv2 [100] to paint all included HM individuals and obtain the co-ancestry matrix. Then, we used fineSTRUCTURE 4.1.0 [100] to dissect the dendrogram based on the haplotype information.

#### Inference of shared IBD segments

We used refined-ibd.17Jan20.102.jar [101] to estimate the shared IBD segments within and between HM individuals. We specified one cM as the minimum length for reported IBD segments (length = 0.1). We used the default values of other parameters, including the sliding marker window of 40.0 and the minimum LOD (logarithm of the odds) score for reported IBD segments of 3. We classified IBD fragments into three classes based on the previously published work: short IBD blocks (1~5 cM), which most likely reflected the ancient genetic connection 1500~2500 years ago; intermediate IBD blocks (5~10 cM), which roundly represented the ancient genetic interaction 500~1500 years ago; and long IBD blocks (> 10 cM), which was likely the result of recent genetic admixture within 500 years [41]. The population size was used to normalize the average shared IBD, and the IBD matrix was visualized as the heatmap or genetic connection in the map.

#### Whole genome sequencing and estimation of effective population size and population split time

To explore the differentiated demographic history of geographically different HM people, we randomly selected two individuals each from SCM, CQM, GZM, GZY, and FJS populations and performed whole-genome sequencing on these samples. The sequencing depth was preset to 10X, and raw data with at least 30 GB was used for subsequent analyses. The raw sequencing reads were aligned to the GRCh37 reference using BWA v.0.7.13 [102], and duplicate reads were marked and removed using the DeDup algorithm implemented in the Sentieon utilities v202010.04 [103]. We then used the Sentieon GVCFtyper algorithm to perform multi-sample variant calling and the Sentieon Haplotyper algorithm to perform haplotype variant calling. We used MSMC2 to estimate the effective population size of each targeted HM-speaking population over time [104]. The Yoruba genome from the 1KGP and the population divergence time between Yoruba and northern Han Chinese were added as a benchmark in the MSMC analysis. We also inferred the population split times between geographically diverse HM-speaking populations and between HM people and northern Han Chinese (Han Chinese in Beijing from the 1KGP)/southern Han Chinese (Han Chinese in South China from the 1KGP) using MSMC2. We estimated the absolute time by assuming a generation time of 29 years and a mutation rate of 1.25 × 10E − 8 per base per generation. We calculated the divergence time between two targeted populations when the rCCR reached 0.5.

#### Identification of natural selection signatures and medical relevance of cancer risk and pharmacogenomic loci

We used the merged Illumina_WGS dataset for the identification of natural selection signals. We regrouped HM-speaking populations based on the estimated genetic affinity and geographical distribution. Here, we separated our HM-related dataset into three panels: the coastal panel included She people from Fujian, inland panel1 included the unique HM genetic structure, and inland panel2 consisted of Miao people from Chongqing. All people showed as outliers or estimated as relatives were removed here. Our primary focus was identifying the natural selection signatures of HM people in the coastal panel and inland panel1. We performed a PBS analysis [105] and adopted the SXH and Europeans from the HGDP as the second and outgroup reference populations to identify HM-specific signatures of local adaptation. We also calculated the PBS values for each HM group using the SXH as the outgroup reference population to identify signatures of natural selection specific to one HM-speaking population. The variants with the top 0.1% of PBS values were selected as the extreme adaptive signals. We conducted additional allele frequency-based (Fst) and haplotype-based (iHS and XPEHH) analyses using Selink (https://github.com/h-e-g/selink) for the detection of classic sweep signals. We retained the top 0.1% of variants in each analysis as candidates for positive selection and assessed the allele frequency distribution of certain candidate loci in worldwide populations from our HM-related dataset, HGDP, and 10K_CPGDP [6, 40]. We then performed GO and KEGG enrichment analysis using Metascape [106]. To assess the value of our HM-related genomic resource in improving human health, we estimated the AFS of known cancer risk loci [5] and pharmacogenomic loci from the ADME core genes (https://www.snpedia.com/index.php/Pharma_ADME) in worldwide populations from the HM-related, NyuWa, 1KGP and GnomAD genomic resources. We did not adjust the sample size when plotting the AFS of medically relevant variants because all meta-populations had sample sizes greater than 30 (except for unrecognized ethnic groups from Guizhou, STM, and CQM, whose sample sizes were close to 30).

### Uniparental population history reconstruction

#### Genotyping and quality control

We extracted 24,047 Y-chromosomal SNPs and 3746 mitochondrial SNPs from the merged Illumina dataset to explore the paternal and maternal population history based on the sharing haplogroups and coalescence processes. We used PLINK v.1.90 to conduct quality control based on the missing SNP and genotyping rates with two parameters (--geno: 0.1 and --mind: 0.1) [107]. In the final quality-control dataset, we retained 11,369 Y-SNPs in 203 individuals and 1428 mtDNA SNPs for uniparental evolutionary history reconstruction.

#### Haplogroup classification, haplogroup frequency spectrum estimation, and clustering analysis

For Y-chromosome haplogroup classification, we used the Python package of hGrpr2.py instrumented in HaploGrouper [108] and the Y-LineageTracker [109] to classify the haplogroups. Two additional reference files were used in the HaploGrouper-based analysis, including the treeFileNEW_isogg2019.txt and snpFile_b38_isogg2019.txt. The Chip version was used in the LineageTracker-based analysis. We also used this software to estimate the haplogroup frequency in different levels of the focused terminal lineages. HaploGrouper and HaploGrep were used to classify the maternal haplogroups.

#### Phylogeny analysis and network analysis

We used the BEAST2.0 [110] and Y-LineageTracker [109] to reconstruct the phylogenetic topology focused on the population divergence, expansion, and migration events. BEAUti, Tracer v1.7.2, and FigTree v1.4.4 were used to prepare the intermediate files for BEAST-based analysis and visualize the resulting phylogeny. The BEAST2.0 was also used to reconstruct the maternal phylogeny. Finally, we used the median-joining Network instructed in the popART [111] to rebuild the network relationship among different haplogroups and populations based on the obtained maternal and paternal genetic variations.

### Supplementary Information

**Additional file 1: Table S1.** The populations newly genotyped in this study and reference populations collected from the Allen Ancient DNA Resource and publicly available databases. **Table S2.** Pairwise Fst values between coastal, inland, Southeast Asian HM people and other reference populations. **Table S3.** Outgroup-*f*_*3*_ values focused on the coastal Hmong-Mien people. **Table S4.** Admixture signatures inferred from the admixture-*f*_*3*_-statistics in the form of *f*_*3*_(source1, source2; targeted populations). **Table S5. **Admixture time estimated using ALDER. **Table S6.** The signatures of natural selection identified in HM people based on Fst, iHS and XPEHH analyses. **Table S7.** The signatures of natural selection specific to Inland HM1 identified based on Fst, iHS and XPEHH analyses. **Table S8.** The signatures of natural selection specific to Coastal HM identified based on Fst, iHS and XPEHH analyses.

**Additional file 2: Fig. S1. **Genetic structure of modern and ancient East Asians. **Fig. S2.** The cross-validation error of model-based ADMIXTURE analysis of 254 modern and ancient populations in the merged Human Origins (HO) dataset. **Fig. S3.** Population admixture and genetic ancestry among 153 ethnolinguistically diverse modern eastern Eurasians and 101 ancient populations from East Asia and surrounding regions. **Fig. S4.** The phylogenetic relationships between geographically diverse HM-speaking populations from China and Southeast Asia revealed by TreeMix analysis with the French as the outgroup population. **Fig. S5.** Model-based ADMIXTURE results of modern and ancient East Asians inferred with predefined ancestral sources ranging from 2 to 11. **Fig. S6.** Model-based ADMIXTURE results of newly genotyped populations and HM-speaking reference populations from China and Southeast Asia inferred with predefined ancestral sources ranging from 2 to 10. **Fig. S7.** A formal test of genomic continuity and admixture in She_Pingshui people inferred from *f*_*4*_-statistics in the form *f*_*4*_(Reference population1, Reference population2; She_Pingshui, Mbuti). **Fig. S8.** A formal test of genomic continuity and admixture in She_Guanshe people inferred from *f*_*4*_-statistics in the form *f*_*4*_(Reference population1, Reference population2; She_Guanshe, Mbuti). **Fig. S9.** A formal test of genomic continuity and admixture in She_Shanyang people inferred from *f*_*4*_-statistics in the form *f*_*4*_(Reference population1, Reference population2; She_Shanyang, Mbuti). **Fig. S10.** A formal test of genomic continuity and admixture in Gaoshan_Huaan people inferred from *f*_*4*_-statistics in the form *f*_*4*_(Reference population1, Reference population2; Gaoshan_Huaan, Mbuti). **Fig. S11.** A formal test of genomic continuity and admixture in She_Guanshe people inferred from *f*_*4*_-statistics in the form *f*_*4*_(Reference population1, She_Guanshe; Reference population2, Mbuti). **Fig. S12.** A formal test of genomic continuity and admixture in She_Pingshui people inferred from *f*_*4*_-statistics in the form *f*_*4*_(Reference population1, She_Pingshui; Reference population2, Mbuti). **Fig. S13.** A formal test of genomic continuity and admixture in She_Shanyang people inferred from *f*_*4*_-statistics in the form *f*_*4*_(Reference population1, She_Shanyang; Reference population2, Mbuti). **Fig. S14.** A formal test of genomic continuity and admixture in Gaoshan_Huaan people inferred from *f*_*4*_-statistics in the form *f*_*4*_(Reference population1, Gaoshan_Huaan; Reference population2, Mbuti). **Fig. S15.** Demographic history of newly genotyped coastal Huaan Gaoshan population. **Fig. S16.** Demographic history of newly genotyped coastal Guanshe She population. **Fig. S17.** Demographic history of newly genotyped coastal Pingshui She population. **Fig. S18.** Demographic history of newly genotyped coastal Shanyang She population. **Fig. S19.** The phylogenetic relationship inferred from paternal and maternal lineages. **Fig. S20.** The effective population sizes (Ne) of inland and coastal HM-speaking populations, and the Yoruba genome was added as a benchmark. The Ne of geographically diverse HM-speaking populations was zoomed in Fig. 3a. **Fig. S21.** The annotation results of candidate signatures of natural selection identified based on PBS, Fst, iHS, and XPEHH approaches. **Fig. S22.** The signatures of natural selection specific to Inland HM1 and Coastal HM were identified based on the PBS approach. **Fig. S23.** The annotation results of candidate genes of natural selection specific to one regional HM-speaking population identified based on Fst, iHS, and XPEHH approaches.

## Data Availability

All data generated or analyzed during this study are included in this published article, its supplementary information files, and publicly available repositories. The genome-wide variation data were collected from the public dataset of Allen Ancient DNA Resource (AADR) (https://reich.hms.harvard.edu/allen-ancient-dna-resource-aadr-downloadable-genotypes-present-day-and-ancient-dna-data). The second-analysis results were submitted in the supplementary materials and also deposited into the OMIX database (https://ngdc.cncb.ac.cn/omix/preview/lJsduf4A) with an accession number of OMIX005474. The raw allele frequency data are available in the Zenodo (https://zenodo.org/records/10453202). The newly reported genotyping data from this study have been deposited into the Zenodo (https://zenodo.org/records/10453218) and Genome Variation Map (https://ngdc.cncb.ac.cn/gvm/home) with an accession number of GVM000631. The acquisition and use of the data shall comply with the regulations of the People’s Republic of China on the administration of human genetic resources. Requests for access to raw data can be directed to Guanglin He (Guanglinhescu@163.com) and Mengge Wang (Menggewang2021@163.com).

## Abbreviations

TB: Tibeto-Burman
HM: Hmong-Mien
TK: Tai-Kadai
AN: Austronesian
AA: Austroasiatic
ChinaMAP: China Metabolic Analytics Project
YRB: Yellow River Basin
YZRB: Yangtze River Basin
ANEA: Ancient Northern East Asian
ASEA: Ancient Southern East Asian
AFS: Allele frequency spectrum
WGS: Whole-genome sequencing
HGDP: Human Genome Diversity Project
1KGP: 1000 Genomes Project
PSS: Pingshui She
GSS: Guanshe She
SYS: Shanyang She
HGDPS: She from the Human Genome Diversity Project
HGDPM: Miao from the Human Genome Diversity Project
HO: Human Origins
AADR: Allen Ancient DNA Resource
PCA: Principal component analysis
CQM: Chongqing Miao
FJS: Fujian She
STM: Songtao Miao
GZM: Guizhou Miao
GZY: Guizhou Yao
HFS: Haplogroup frequency spectrum
rCCR: Relative cross coalescence rate
IBD: Identity-by-descent
ROH: Runs of Homozygosity
ADME: Absorption, distribution, metabolism, and excretion
PBS: Population branch statistics
iHS: Integrated haplotype score
XPEHH: Cross-population extended haplotype homozygosity
SXH: Han_Shaanxi
10K_CPGDP: 10K Chinese People Genomic Diversity Project
CCHCR1: Coiled-coil alpha-helical rod protein 1
HDVs: Highly differentiated variants
CR1: Complement receptor 1
TRIM31: Tripartite motif containing 31
GPCR: G protein-coupled receptor
GO: Gene Ontology
KEGG: Kyoto Encyclopedia of Genes and Genomes
WBBC: Westlake BioBank for Chinese
SHAPEIT: Segmented HAPlotype Estimation & Imputation Tool
