# Supplementary material for: Differentiated genomic footprints suggest isolation and long-distance migration of Hmong-Mien populations
Source: BMC Biol. 2024 Jan 25;22:18. doi: 10.1186/s12915-024-01828-x (PMC10809681; doi:10.1186/s12915-024-01828-x)
Supplement: Supplementary file 2 — Additional file 2: Fig. S1. Genetic structure of modern and ancient East Asians. Fig. S2. The cross-validation error of model-based ADMIXTURE analysis of 254 modern and ancient populations in the merged Human Origins (HO) dataset. Fig. S3. Population admixture and genetic ancestry among 153 ethnolinguistically diverse modern eastern Eurasians and 101 ancient populations from East Asia and surrounding regions. Fig. S4. The phylogenetic relationships between geographically diverse HM-speaking populations from China and Southeast Asia revealed by TreeMix analysis with the French as the outgroup population. Fig. S5. Model-based ADMIXTURE results of modern and ancient East Asians inferred with predefined ancestral sources ranging from 2 to 11. Fig. S6. Model-based ADMIXTURE results of newly genotyped populations and HM-speaking reference populations from China and Southeast Asia inferred with predefined ancestral sources ranging from 2 to 10. Fig. S7. A formal test of genomic continuity and admixture in She_Pingshui people inferred from f4-statistics in the form f4(Reference population1, Reference population2; She_Pingshui, Mbuti). Fig. S8. A formal test of genomic continuity and admixture in She_Guanshe people inferred from f4-statistics in the form f4(Reference population1, Reference population2; She_Guanshe, Mbuti). Fig. S9. A formal test of genomic continuity and admixture in She_Shanyang people inferred from f4-statistics in the form f4(Reference population1, Reference population2; She_Shanyang, Mbuti). Fig. S10. A formal test of genomic continuity and admixture in Gaoshan_Huaan people inferred from f4-statistics in the form f4(Reference population1, Reference population2; Gaoshan_Huaan, Mbuti). Fig. S11. A formal test of genomic continuity and admixture in She_Guanshe people inferred from f4-statistics in the form f4(Reference population1, She_Guanshe; Reference population2, Mbuti). Fig. S12. A formal test of genomic continuity and admixture in She_Pingshui people [file 12915_2024_1828_MOESM2_ESM.pdf]

Supplementary Figures

RESEARCH Open

Differentiated genomic footprints suggest isolation and long-distance migration of Hmong-Mien populations

Guanglin He[1,2,6,#,*] , Peixin Wang[1,4], Jing Chen[1,5], Yan Liu[1,6], Yuntao Sun[1,7], Rong Hu[8], Shuhan Duan[1,6], Qiuxia Sun[1,9], Renkuan Tang[9], Junbao Yang[6], Zhiyong Wang[1,10], Libing Yun[7], Liping Hu[1,10], Jiangwei Yan[5], Shengjie Nie[10], Lanhai Wei[11,*], Chao Liu[3,12,13,#], Mengge Wang[1,2,3,#,*]

Correspondence: Guanglin He (guanglinhescu@163.com), Chao Liu (liuchaogzf@163.com), Mengge Wang (Menggewang2021@163.com)
*Guanglin He, Lanhai Wei and Mengge Wang contributed equally to this work.
[1]Institute of Rare Diseases, West China Hospital of Sichuan University, Sichuan University, Chengdu, 610041, China
[2]Center for Archaeological Science, Sichuan University, Chengdu, 610000, China
[3]Faculty of Forensic Medicine, Zhongshan School of Medicine, Sun Yat-sen University, Guangzhou, 510275, China
[4]School of Medical Information, Chongqing Medical University, Chongqing, 400331, China
[5]School of Forensic Medicine, Shanxi Medical University, Jinzhong, 030001, China
[6]School of Basic Medical Sciences, North Sichuan Medical College, Nanchong, 637000, China
[7]Institute of Forensic Medicine, West China School of Basic Science & Forensic Medicine, Sichuan University, Chengdu, 610041, China
[8]School of Sociology and Anthropology, Xiamen University, Xiamen, 361005, China
[9]Department of Forensic Medicine, College of Basic Medicine, Chongqing Medical University, Chongqing, 400331, China
[10]School of Forensic Medicine, Kunming Medical University, Kunming, 650500, China
[11]School of Ethnology and Anthropology, Inner Mongolia Normal University, Inner Mongolia, 010028, China
[12]Anti-Drug Technology Center of Guangdong Province, Guangzhou, 510230, China
[13]Guangzhou Key Laboratory of Forensic Multi-Omics for Precision Identification, School of Forensic Medicine, Southern Medical University, Guangzhou, 510515, China

Contents

Figure S1. Genetic structure of modern and ancient East Asians. 4

Figure S2. The cross-validation error of model-based ADMIXTURE analysis of 254 modern and ancient populations in the merged Human Origins (HO) dataset. 4

Figure S3. Population admixture and genetic ancestry among 153 ethnolinguistically diverse modern eastern Eurasians and 101 ancient populations from East Asia and surrounding regions. 5

Figure S4. The phylogenetic relationships between geographically diverse HM-speaking populations from China and Southeast Asia revealed by TreeMix analysis with the French as the outgroup population. 5

Figure S5. Model-based ADMIXTURE results of modern and ancient East Asians inferred with predefined ancestral sources ranging from 2 to 11. 6

Figure S6. Model-based ADMIXTURE results of newly genotyped populations and HM-speaking reference populations from China and Southeast Asia inferred with predefined ancestral sources ranging from 2 to 10. 6

Figure S7. A formal test of genomic continuity and admixture in She_Pingshui people inferred from f_4-statistics in the form f_4*(Reference population1, Reference population2; She_Pingshui, Mbuti)*. 7

Figure S8. A formal test of genomic continuity and admixture in She_Guanshe people inferred from f_4-statistics in the form f_4*(Reference population1, Reference population2; She_Guanshe, Mbuti)*. 8

Figure S9. A formal test of genomic continuity and admixture in She_Shanyang people inferred from f_4-statistics in the form f_4*(Reference population1, Reference population2; She_Shanyang, Mbuti)*. 9

Figure S10. A formal test of genomic continuity and admixture in Gaoshan_Huaan people inferred from f_4-statistics in the form f_4*(Reference population1, Reference population2; Gaoshan_Huaan, Mbuti)*. 10

Figure S11. A formal test of genomic continuity and admixture in She_Guanshe people inferred from f_4-statistics in the form f_4*(Reference population1, She_Guanshe; Reference population2, Mbuti)*. 11

Figure S12. A formal test of genomic continuity and admixture in She_Pingshui people inferred from f_4-statistics in the form f_4*(Reference population1, She_Pingshui; Reference population2, Mbuti)*. 12

Figure S13. A formal test of genomic continuity and admixture in She_Shanyang people inferred from f_4-statistics in the form f_4*(Reference population1, She_Shanyang; Reference population2, Mbuti)*. 13

Figure S14. A formal test of genomic continuity and admixture in Gaoshan_Huaan people inferred from f_4-statistics in the form f_4*(Reference population1, Gaoshan_Huaan; Reference population2, Mbuti)*. 14

Figure S15. Demographic history of newly genotyped coastal Huaan Gaoshan population. 15

Figure S16. Demographic history of newly genotyped coastal Guanshe She population. 16

Figure S17. Demographic history of newly genotyped coastal Pingshui She population. 17

Figure S18. Demographic history of newly genotyped coastal Shanyang She population. 18

Figure S19. The phylogenetic relationship inferred from paternal and maternal lineages. 19

Figure S20. The effective population sizes (Ne) of inland and coastal HM-speaking populations, and the

Yoruba genome was added as a benchmark. The Ne of geographically diverse HM-speaking populations was zoomed in Figure 3a. 20

Figure S21. The annotation results of candidate signatures of natural selection identified based on PBS, Fst, iHS, and XPEHH approaches. 20

Figure S22. The signatures of natural selection specific to Inland HM1 and Coastal HM were identified based on the PBS approach. 21

Figure S23. The annotation results of candidate genes of natural selection specific to one regional HM-speaking population identified based on Fst, iHS, and XPEHH approaches. 21

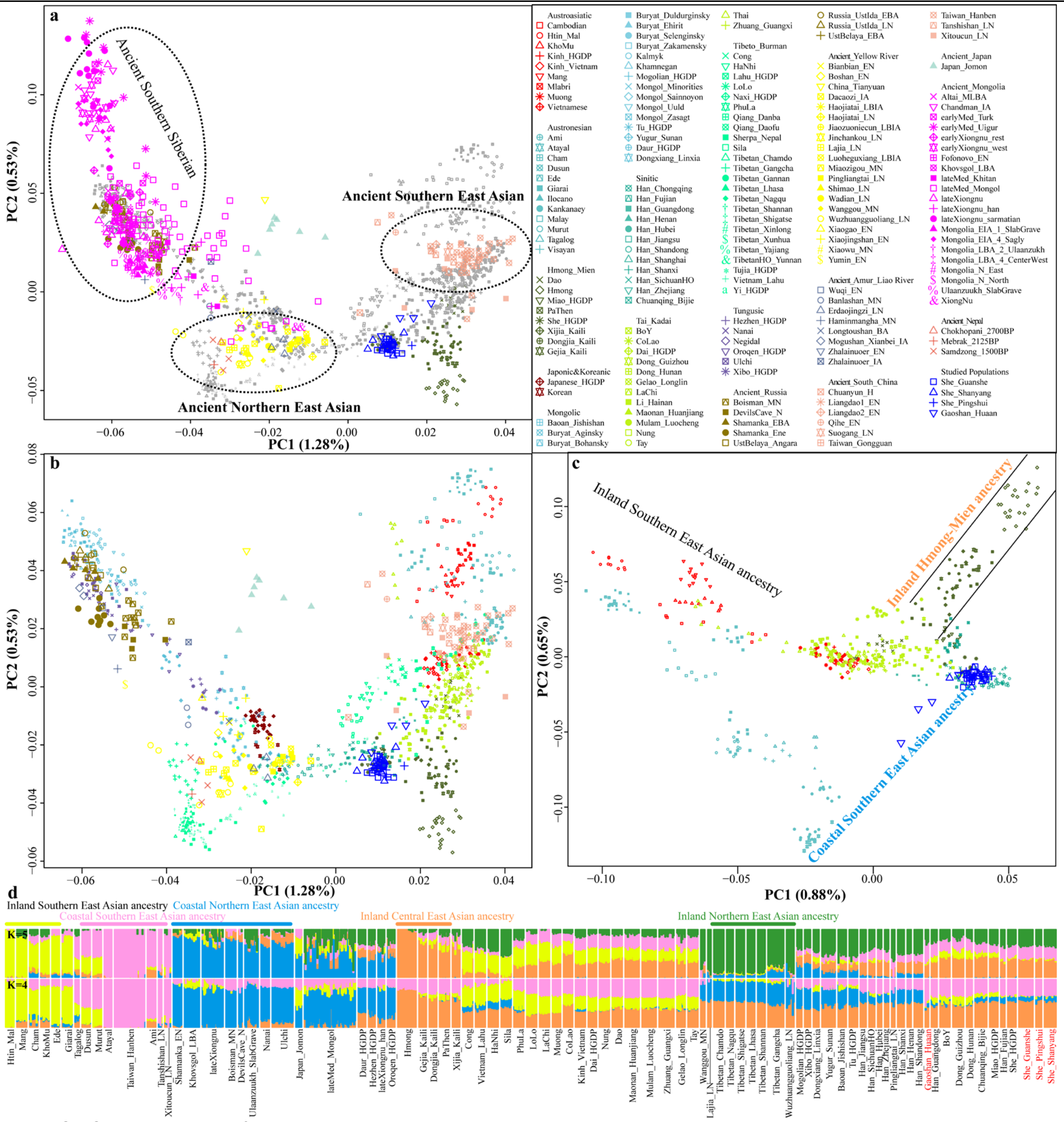

Figure S1. Genetic structure of modern and ancient East Asians.
(**a**) The results of principal component analysis (PCA) in the context of modern and ancient eastern Eurasians. We projected ancient populations into the top two coordinates extracted via modern populations, and present-day individuals were shown in gray. (**b**) The genetic relationship between modern and ancient East and Southeast Asians except ancient southern Siberians. (**c**) Population structure of modern and ancient southern East Asians and Southeast Asians. (**d**) Model-based ADMIXTURE results of modern and ancient East Asians inferred with predefined ancestral sources ranging from 4 to 5.

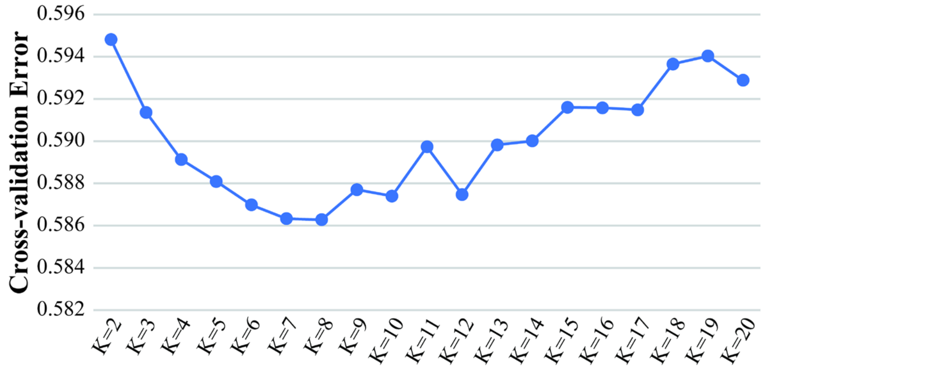

Figure S2. The cross-validation error of model-based ADMIXTURE analysis of 254 modern and ancient populations in the merged Human Origins (HO) dataset.

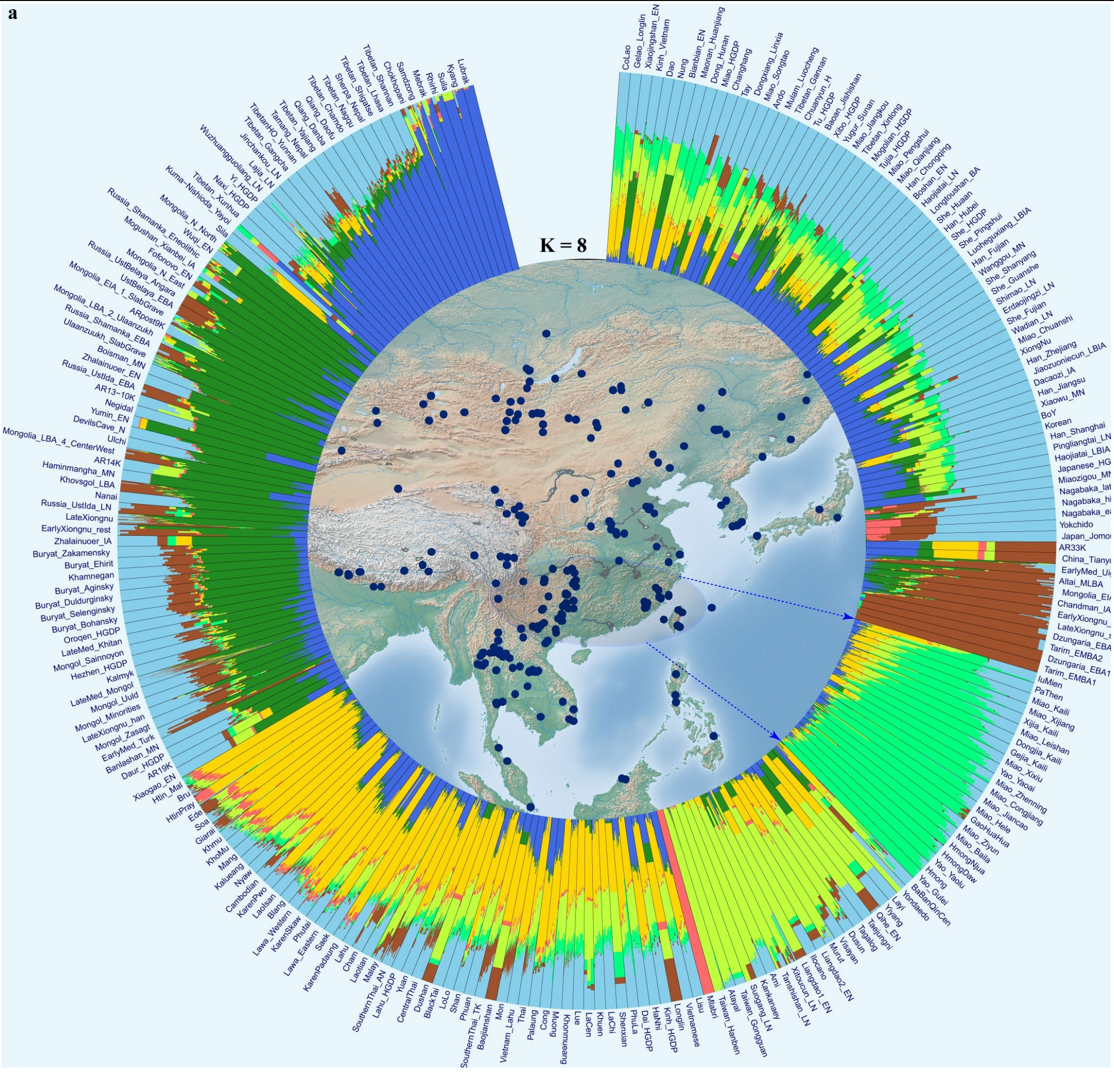

Figure S3. Population admixture and genetic ancestry among 153 ethnolinguistically diverse modern eastern Eurasians and 101 ancient populations from East Asia and surrounding regions.
The ancestral composition of 254 modern and ancient populations was inferred based on the genetic variations in the merged HO dataset. The cross-validation error in **Figure S2** indicated that the model with eight ancestral sources was the best-fitted model. The geographical distribution of all included modern and ancient populations was shown on the central map, with detailed latitudes and longitudes for each group presented in **Table S1**.

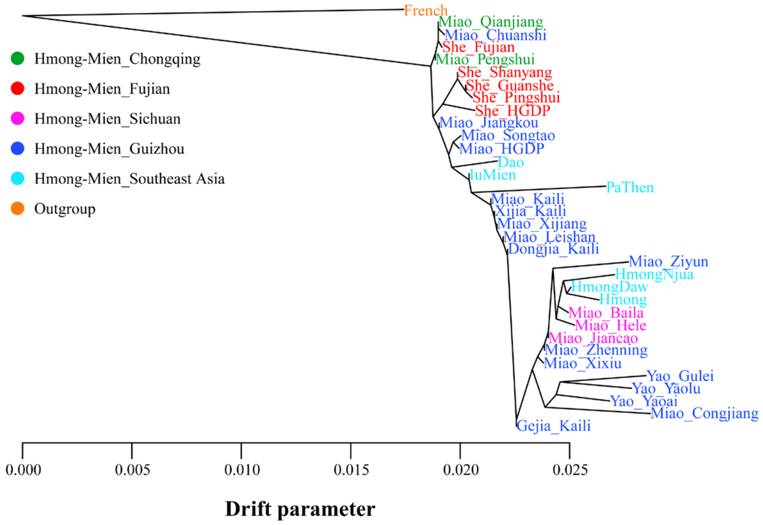

Figure S4. The phylogenetic relationships between geographically diverse HM-speaking populations from China and Southeast Asia revealed by TreeMix analysis with the French as the outgroup population.

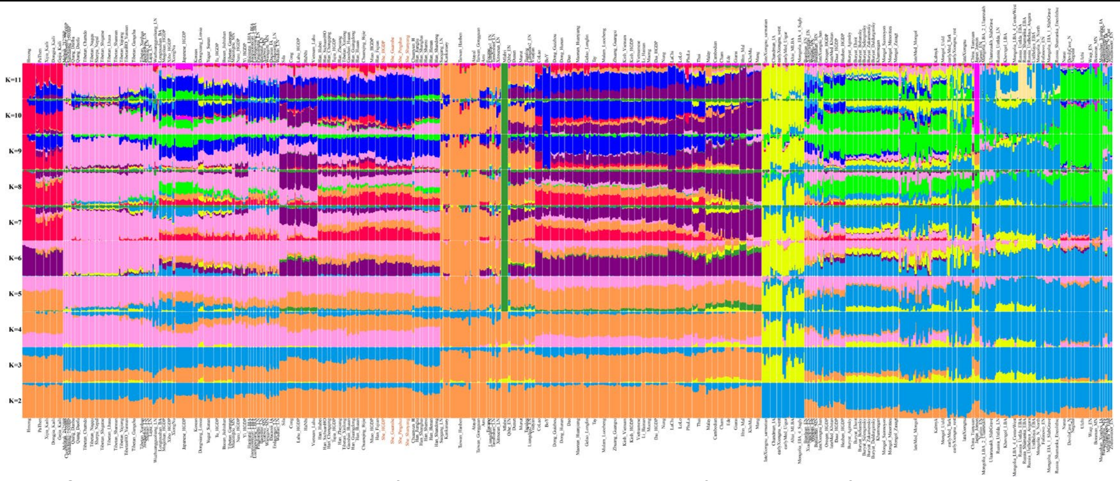

Figure S5. Model-based ADMIXTURE results of modern and ancient East Asians inferred with predefined ancestral sources ranging from 2 to 11.

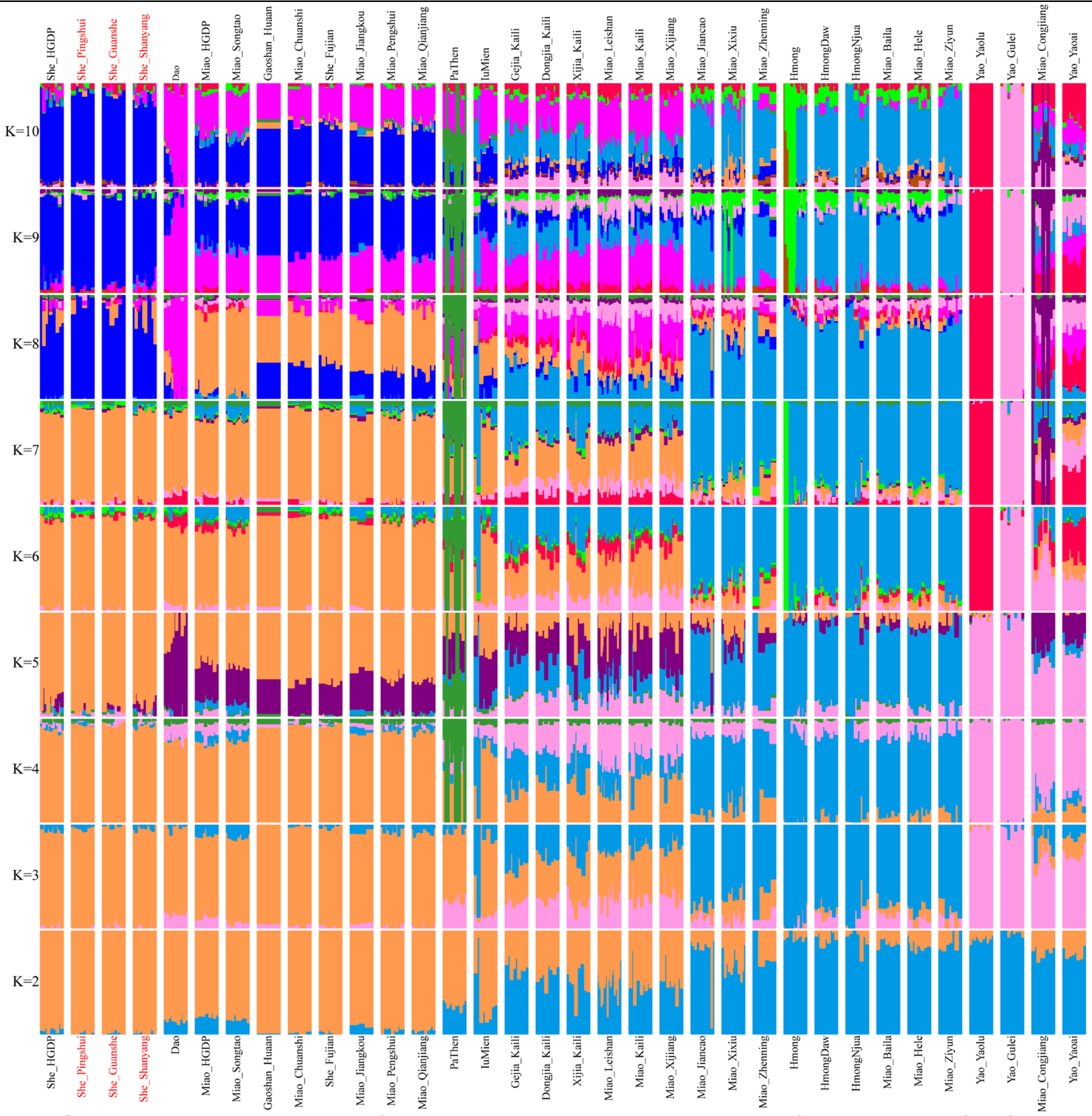

Figure S6. Model-based ADMIXTURE results of newly genotyped populations and HM-speaking reference populations from China and Southeast Asia inferred with predefined ancestral sources ranging from 2 to 10.

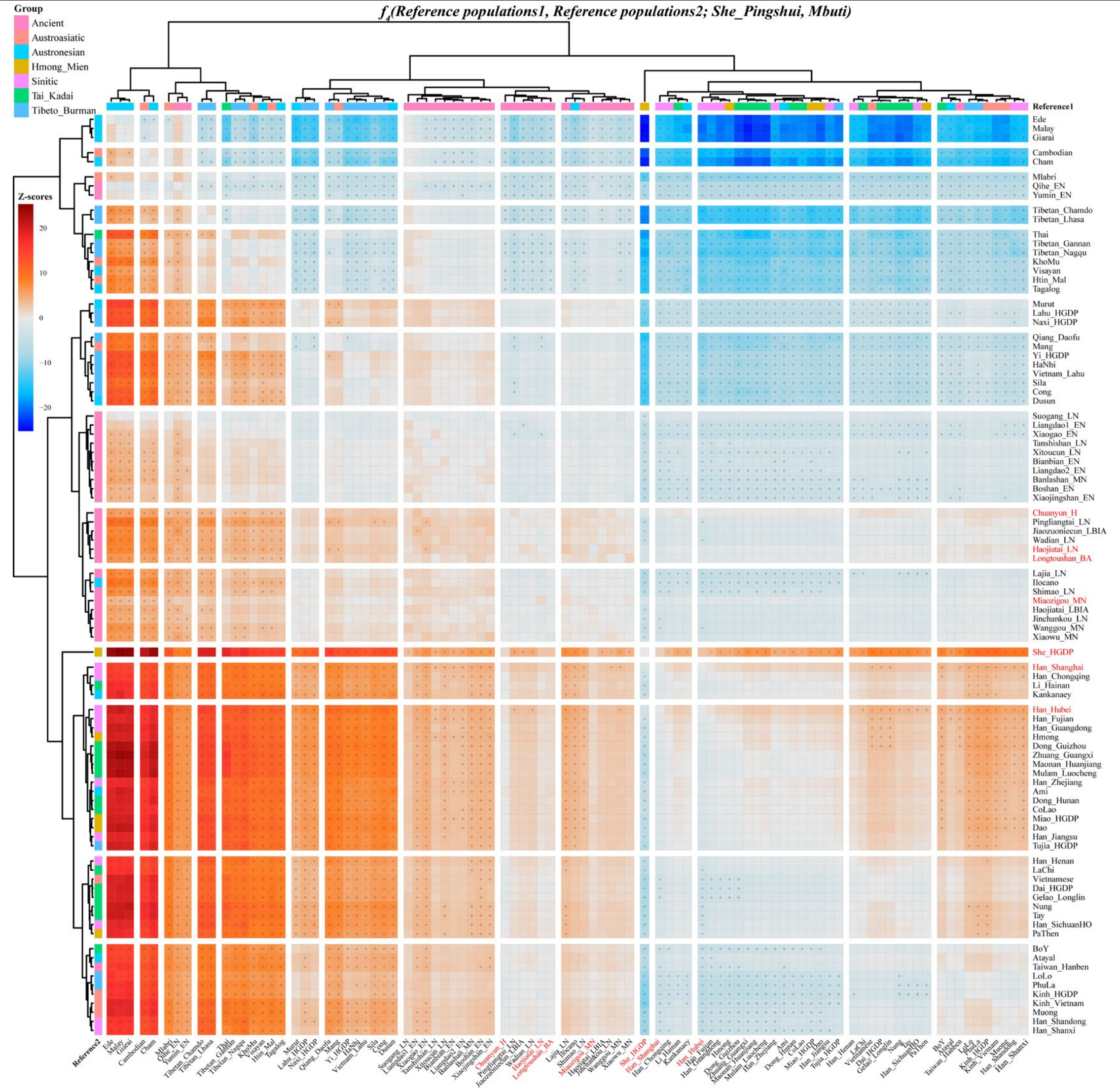

Figure S7. A formal test of genomic continuity and admixture in She_Pingshui people inferred from f_4-statistics in the form *f_4(Reference population1, Reference population2; She_Pingshui, Mbuti)*.

The red color denoted the positive f_4-values, which suggested that compared with reference population2 (bottom population lists), She_Pingshui shared more derived mutations with reference population1 (right population lists), the blue color showed the negative f_4-values, which suggested that reference population2 shared more alleles with She_Pingshui relative to reference population1, and the gray color showed no statistically significant results. Statistically significant results were marked with the '+'.

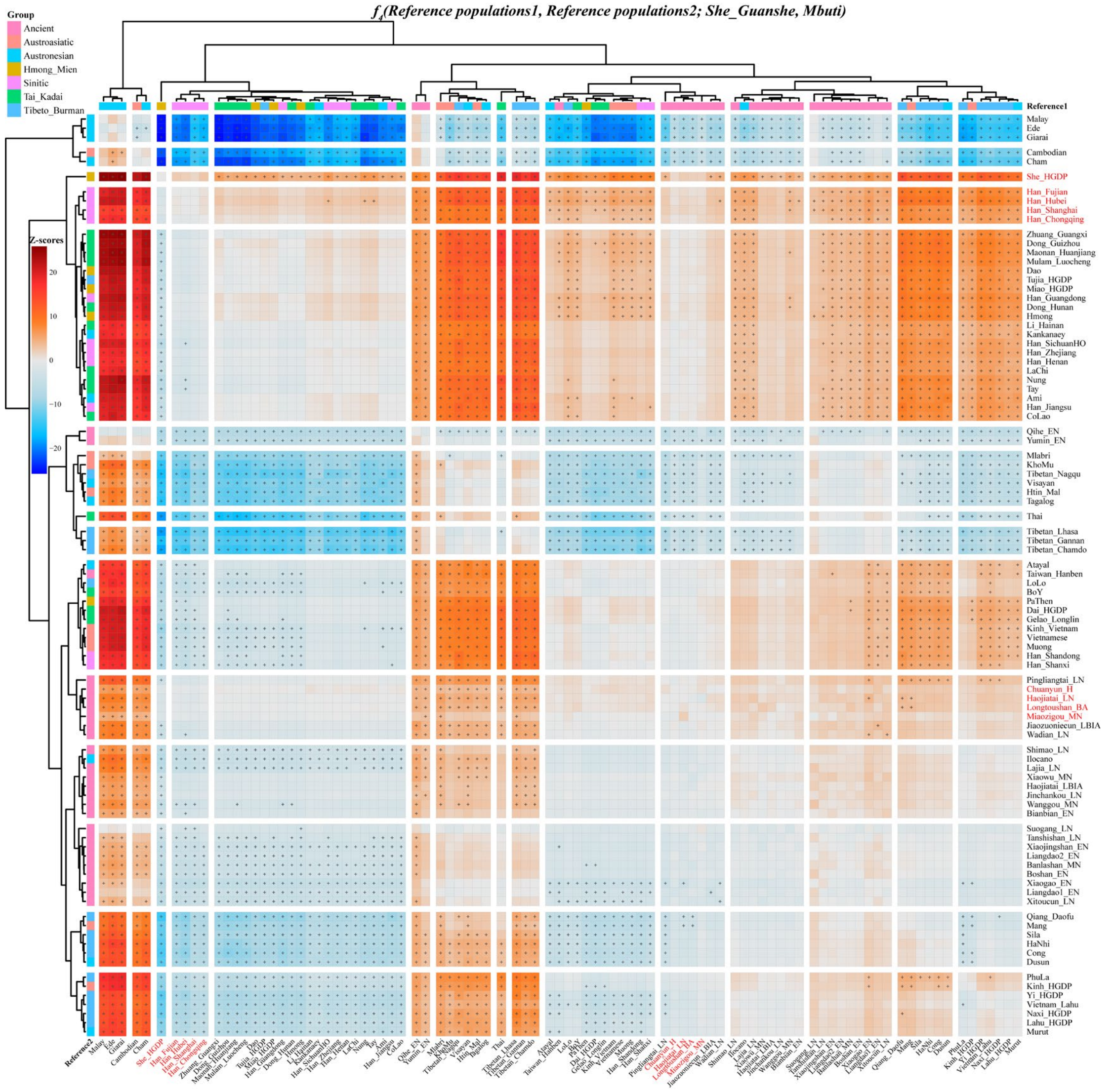

Figure S8. A formal test of genomic continuity and admixture in She_Guanshe people inferred from f_4-statistics in the form f_4*(Reference population1, Reference population2; She_Guanshe, Mbuti)*.

The red color denoted the positive f_4-values, which suggested that compared with reference population2 (bottom population lists), She_Guanshe shared more derived mutations with reference population1 (right population lists), the blue color showed the negative f_4-values, which suggested that reference population2 shared more alleles with She_Guanshe relative to reference population1, and the gray color showed no statistically significant results. Statistically significant results were marked with the '+'.

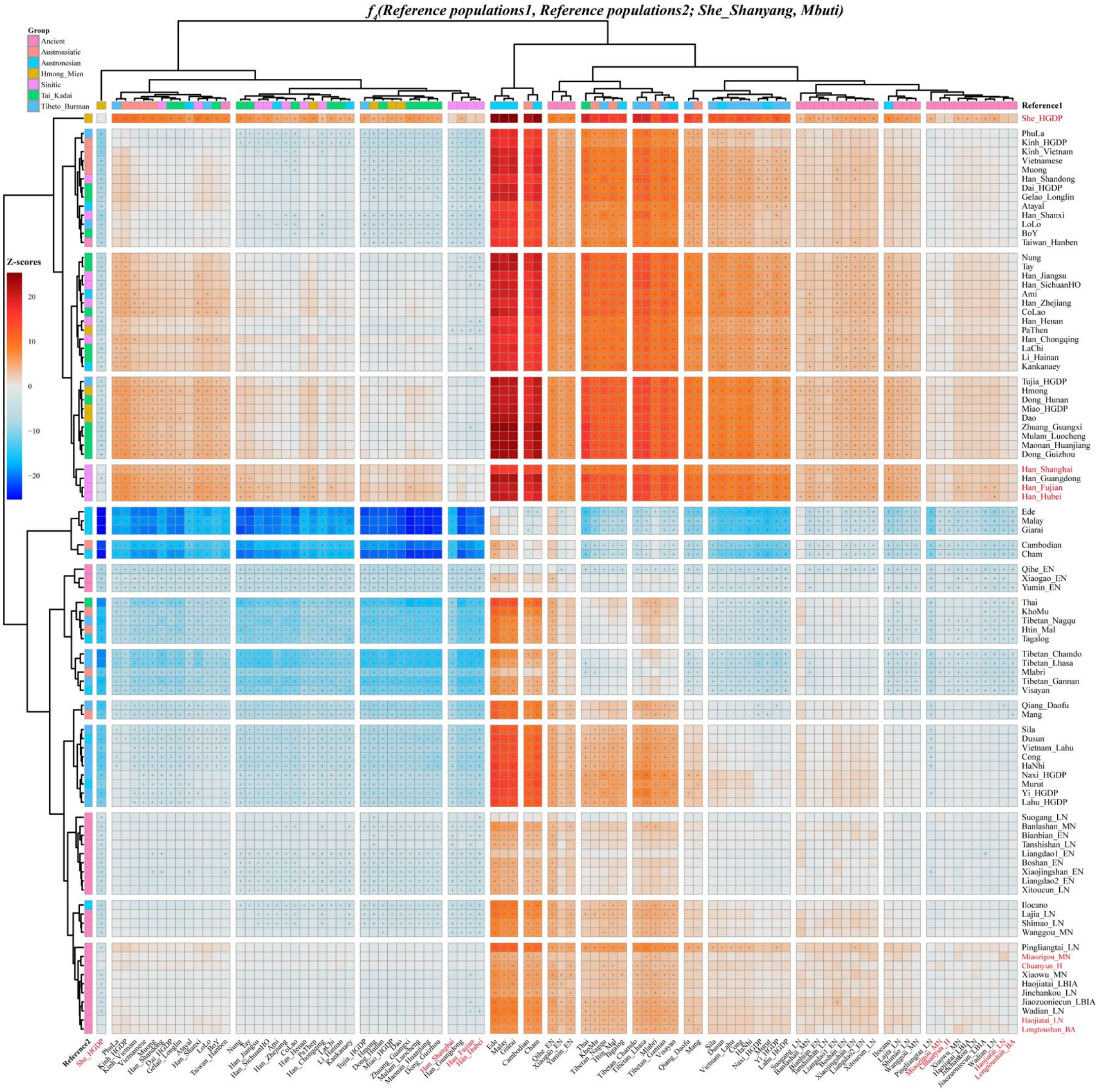

Figure S9. A formal test of genomic continuity and admixture in She_Shanyang people inferred from f_4-statistics in the form f_4(Reference population1, Reference population2; She_Shanyang, Mbuti).

The red color denoted the positive f_4-values, which suggested that compared with reference population2 (bottom population lists), She_Shanyang shared more derived mutations with reference population1 (right population lists), the blue color showed the negative f_4-values, which suggested that reference population2 shared more alleles with She_Shanyang relative to reference population1, and the gray color showed no statistically significant results. Statistically significant results were marked with the '+'.

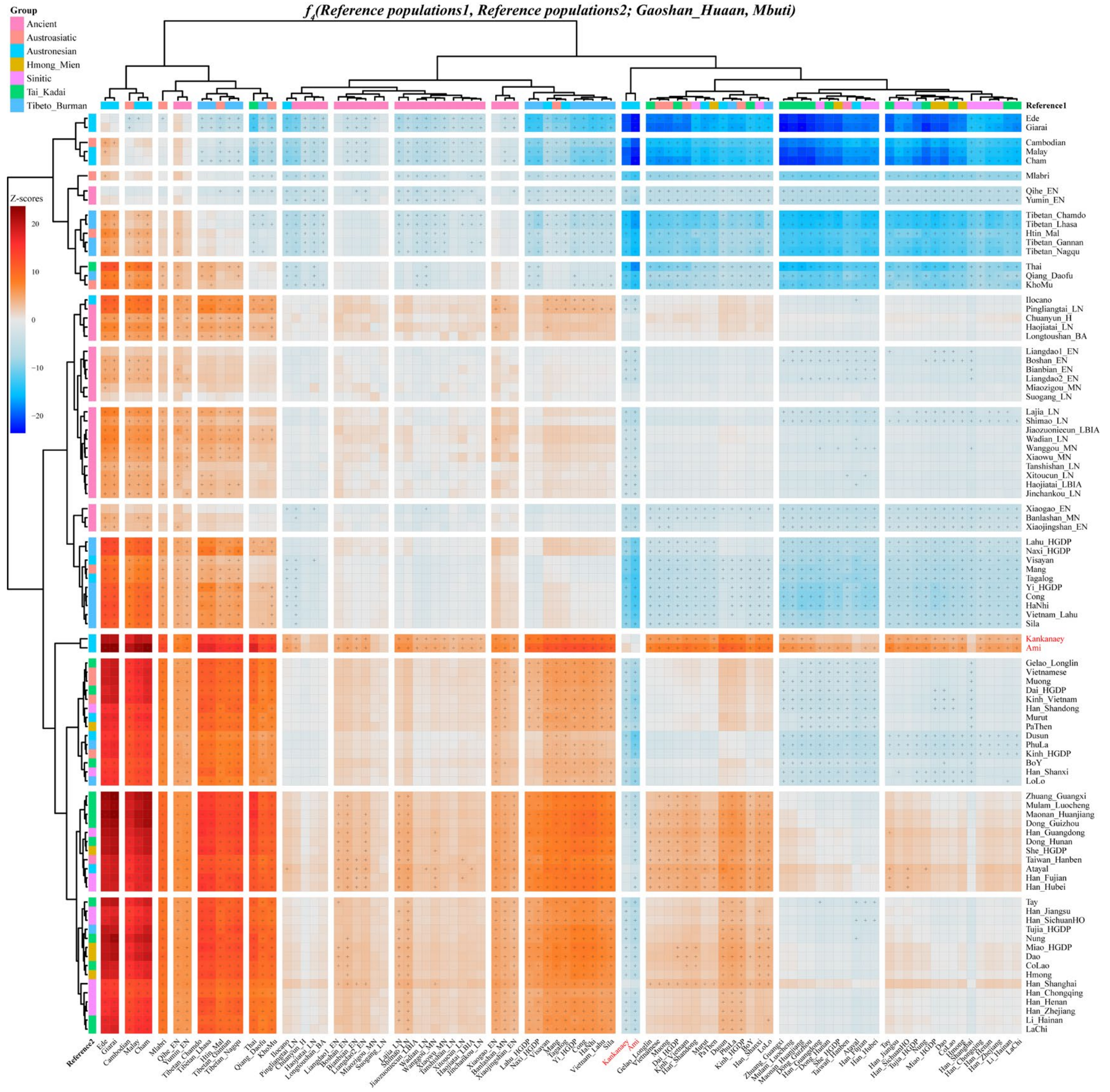

Figure S10. A formal test of genomic continuity and admixture in Gaoshan_Huaan people inferred from f_4-statistics in the form f_4*(Reference population1, Reference population2; Gaoshan_Huaan, Mbuti)*.

The red color denoted the positive f_4-values, which suggested that compared with reference population2 (bottom population lists), Gaoshan_Huaan shared more derived mutations with reference population1 (right population lists), the blue color showed the negative f_4-values, which suggested that reference population2 shared more alleles with Gaoshan_Huaan relative to reference population1, and the gray color showed no statistically significant results. Statistically significant results were marked with the '+'.

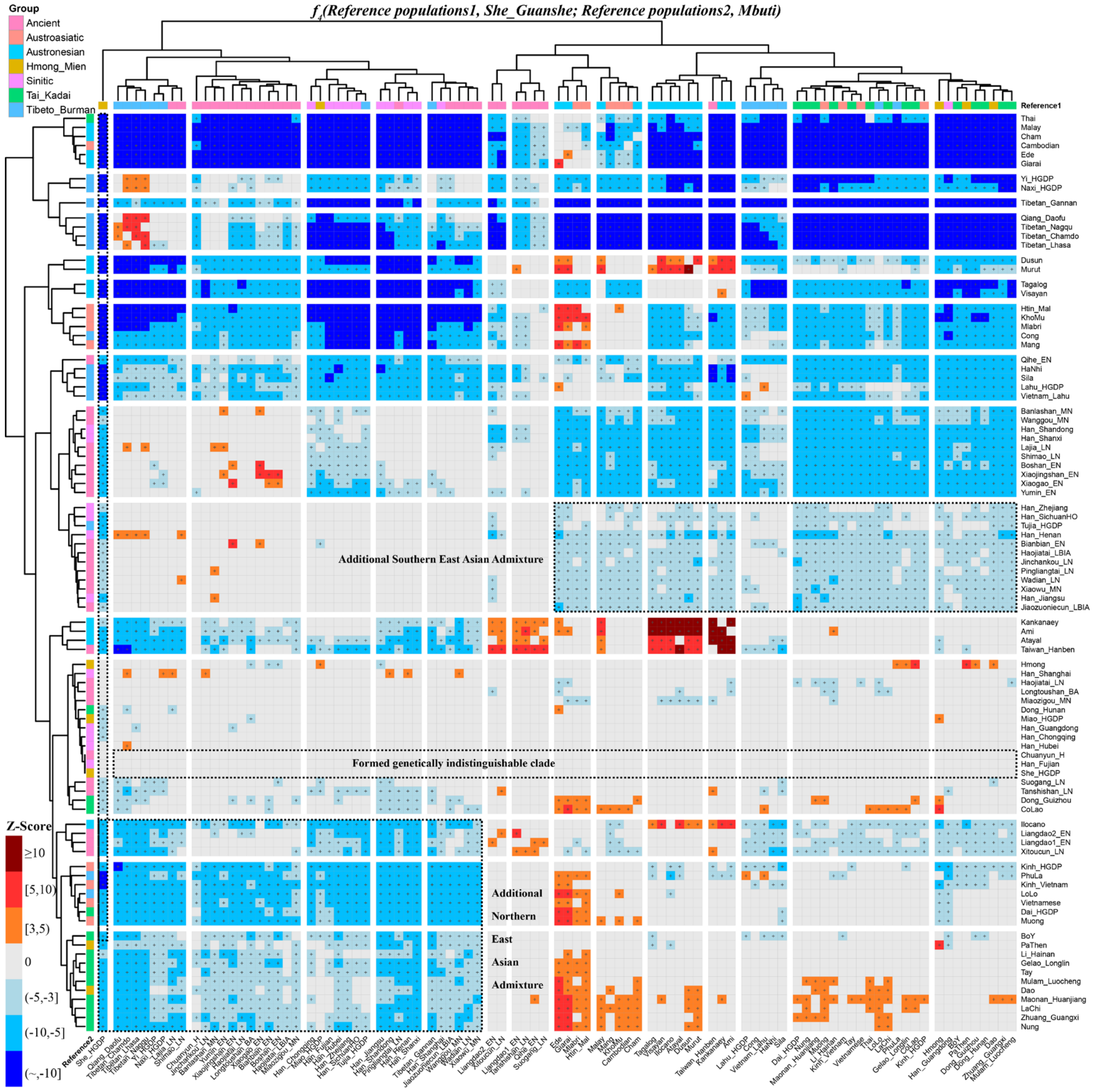

Figure S11. A formal test of genomic continuity and admixture in She_Guanshe people inferred from f_4-statistics in the form f_4(Reference population1, She_Guanshe; Reference population2, Mbuti).

The red color denoted the positive f_4-values, which suggested that Reference population2 (bottom population lists) shared more derived mutations with reference population1 (right population lists), the blue color showed the negative f_4-values, which suggested that reference population2 shared more alleles with She_Guanshe, and the gray color showed no statistically significant results. Statistically significant results were marked with the '+'.

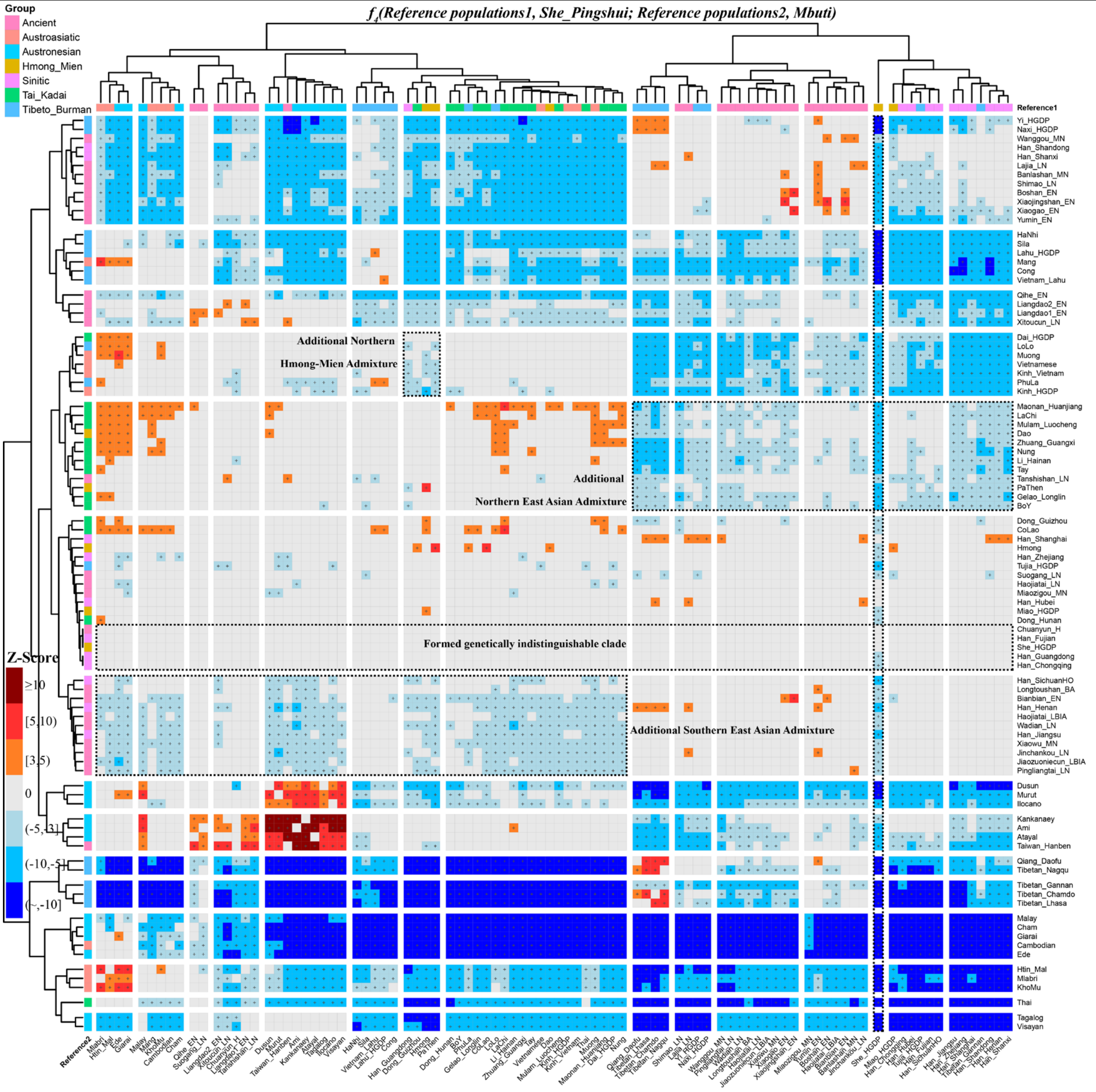

Figure S12. A formal test of genomic continuity and admixture in She_Pingshui people inferred from f_4-statistics in the form f_4(Reference population1, She_Pingshui; Reference population2, Mbuti).

The red color denoted the positive f_4-values, which suggested that Reference population2 (bottom population lists) shared more derived mutations with reference population1 (right population lists), the blue color showed the negative f_4-values, which suggested that reference population2 shared more alleles with She_Pingshui, and the gray color showed no statistically significant results. Statistically significant results were marked with the '+'.

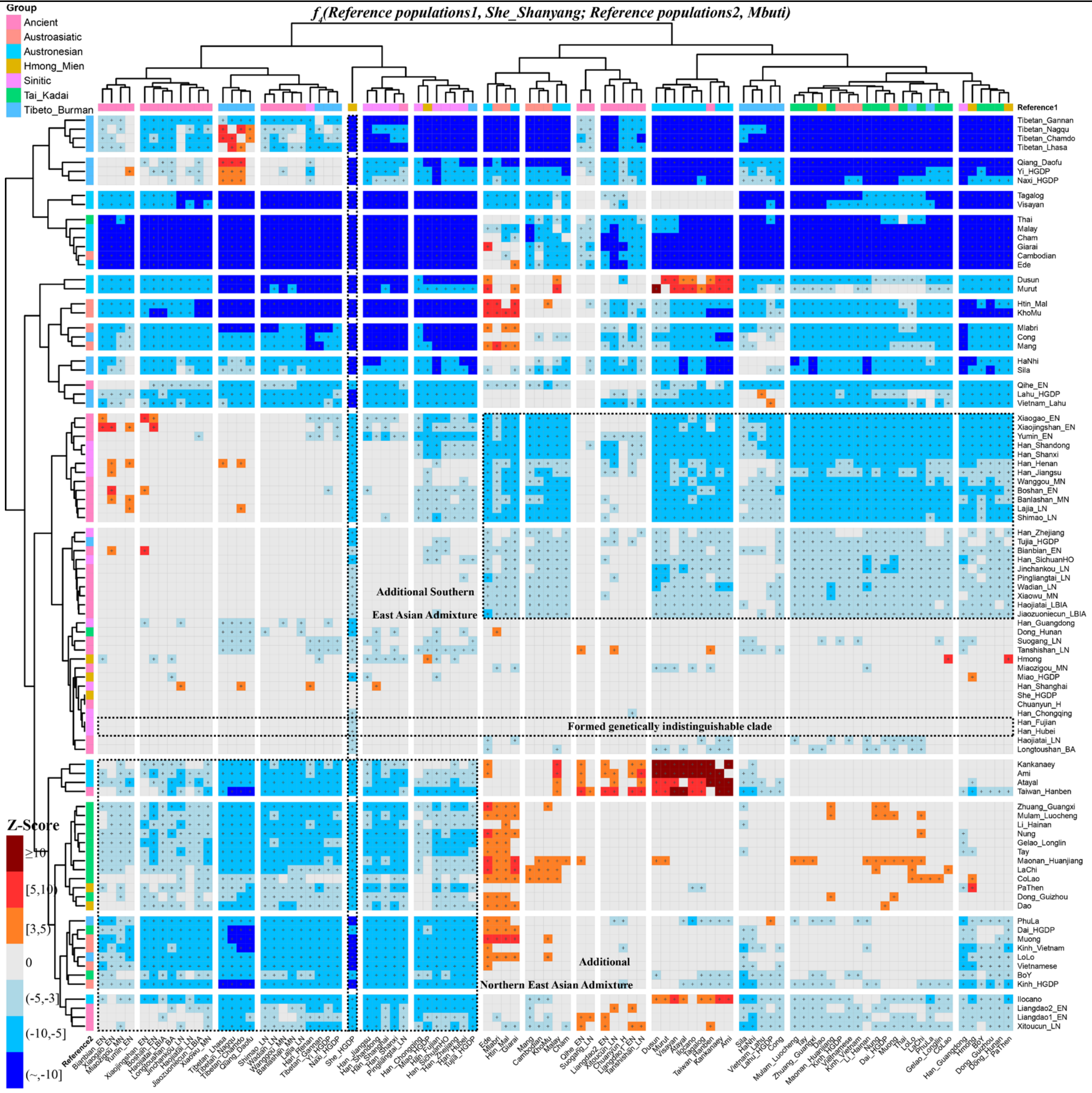

Figure S13. A formal test of genomic continuity and admixture in She_Shanyang people inferred from f_4-statistics in the form f_4(Reference population1, She_Shanyang; Reference population2, Mbuti).

The red color denoted the positive f_4-values, which suggested that Reference population2 (bottom population lists) shared more derived mutations with reference population1 (right population lists), the blue color showed the negative f_4-values, which suggested that reference population2 shared more alleles with She_Shanyang, and the gray color showed no statistically significant results. Statistically significant results were marked with the '+'.

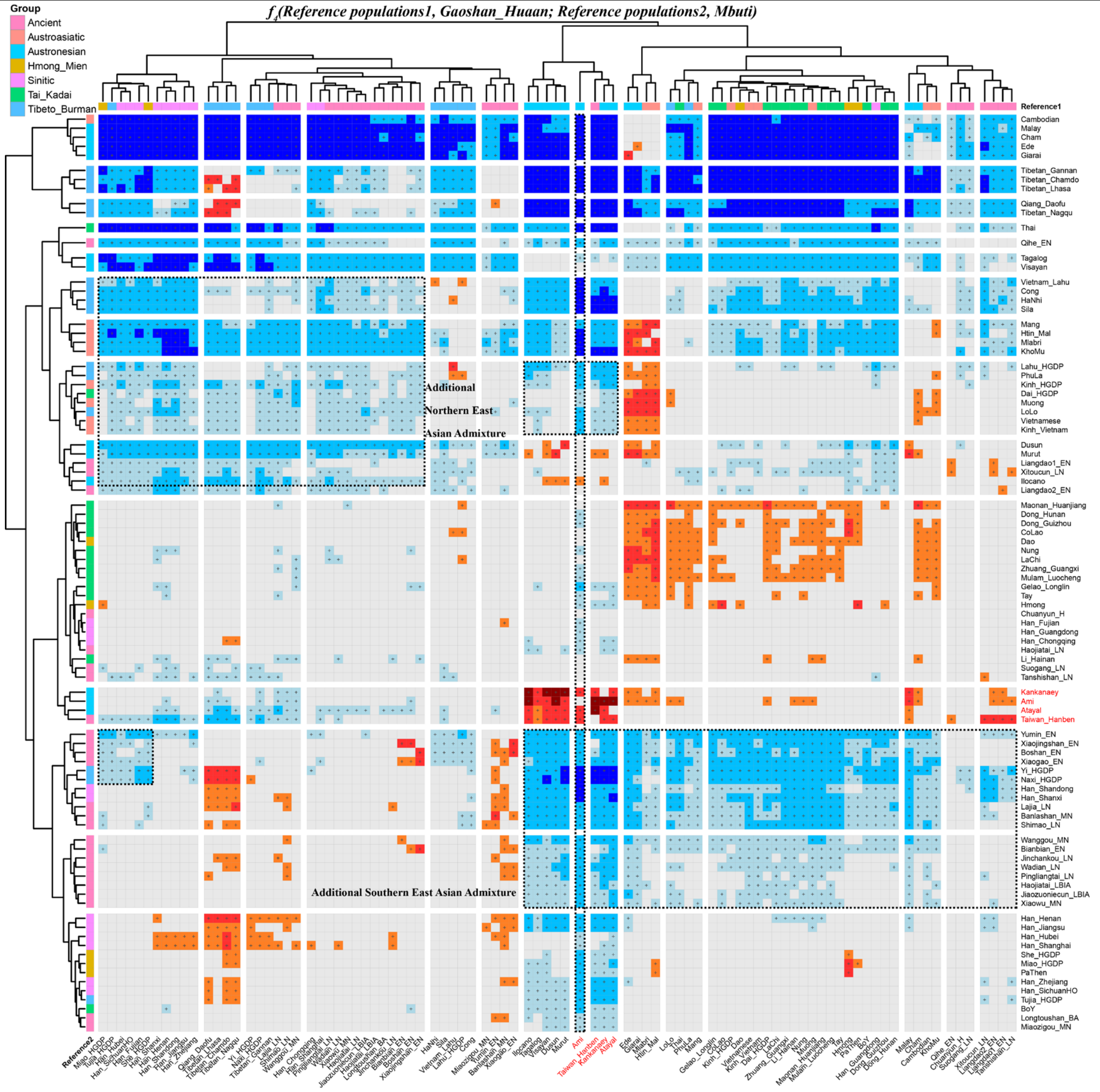

Figure S14. A formal test of genomic continuity and admixture in Gaoshan_Huaan people inferred from f_4-statistics in the form f_4(Reference population1, Gaoshan_Huaan; Reference population2, Mbuti).

The red color denoted the positive f_4-values, which suggested that Reference population2 (bottom population lists) shared more derived mutations with reference population1 (right population lists), the blue color showed the negative f_4-values, which suggested that reference population2 shared more alleles with Gaoshan_Huaan, and the gray color showed no statistically significant results. Statistically significant results were marked with the '+'.

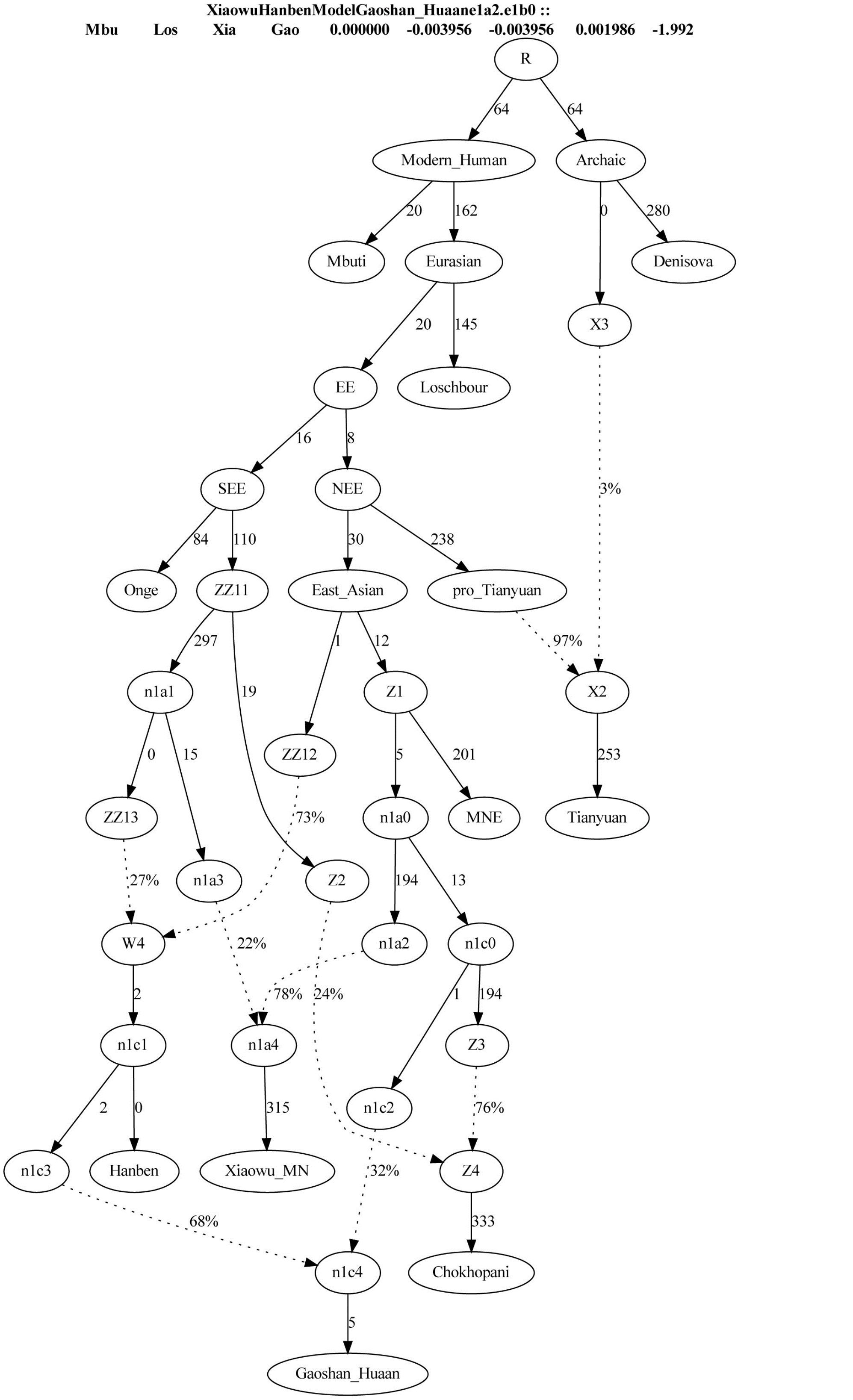

Figure S15. Demographic history of newly genotyped coastal Huaan Gaoshan population.
The presented qpGraph-based phylogenetic topology was fitted for Gaoshan people with the best-worst Z-score of -1.992. Branch lengths were labeled in units of f_2 genetic drift distance times 1000. The dotted line indicates the admixture events with admixture proportion.

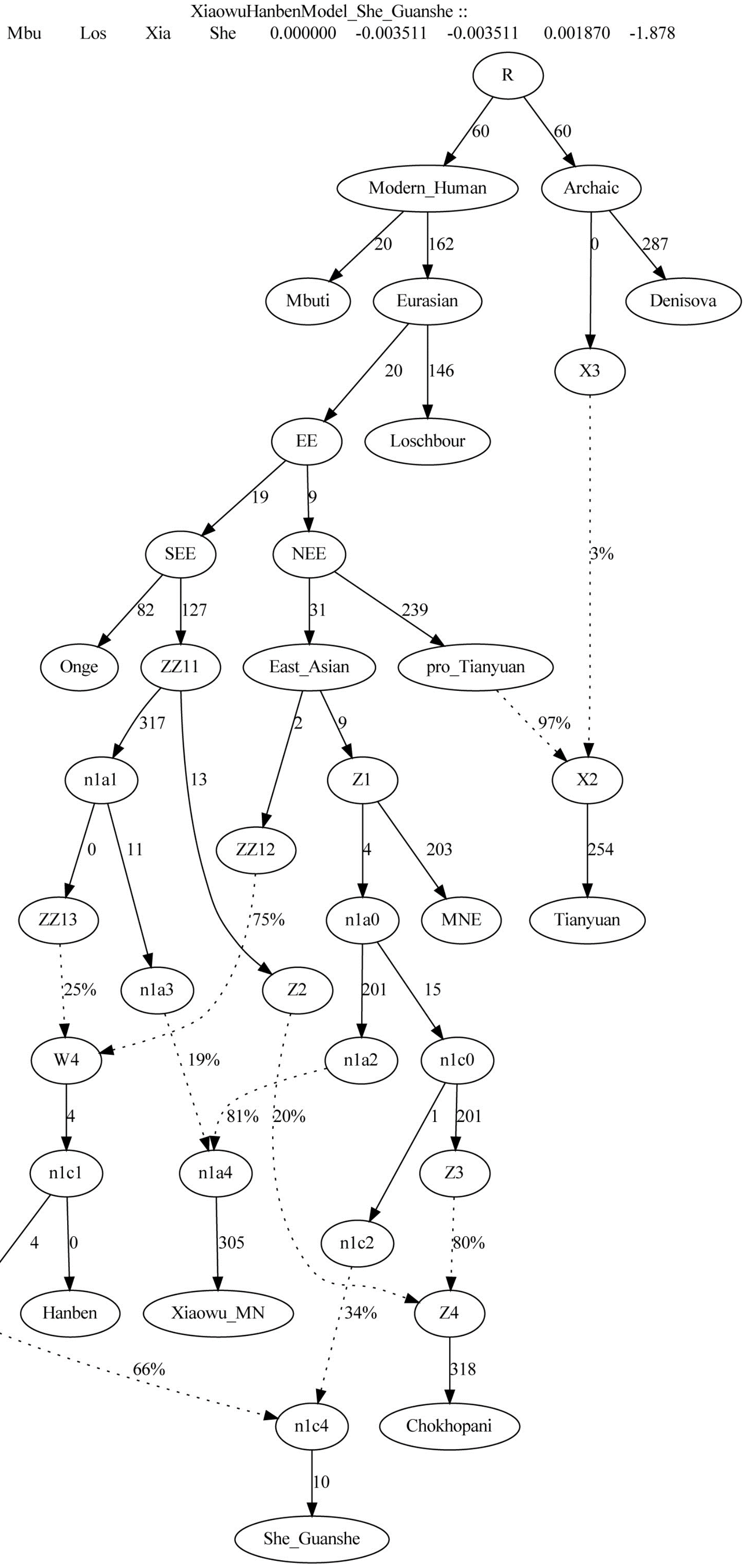

Figure S16. Demographic history of newly genotyped coastal Guanshe She population.
The presented qpGraph-based phylogenetic topology was fitted for Guanshe She people with the best-worst Z-score of -1.878. Branch lengths were labeled in units of f_2 genetic drift distance times 1000. The dotted line indicates the admixture events with admixture proportion.

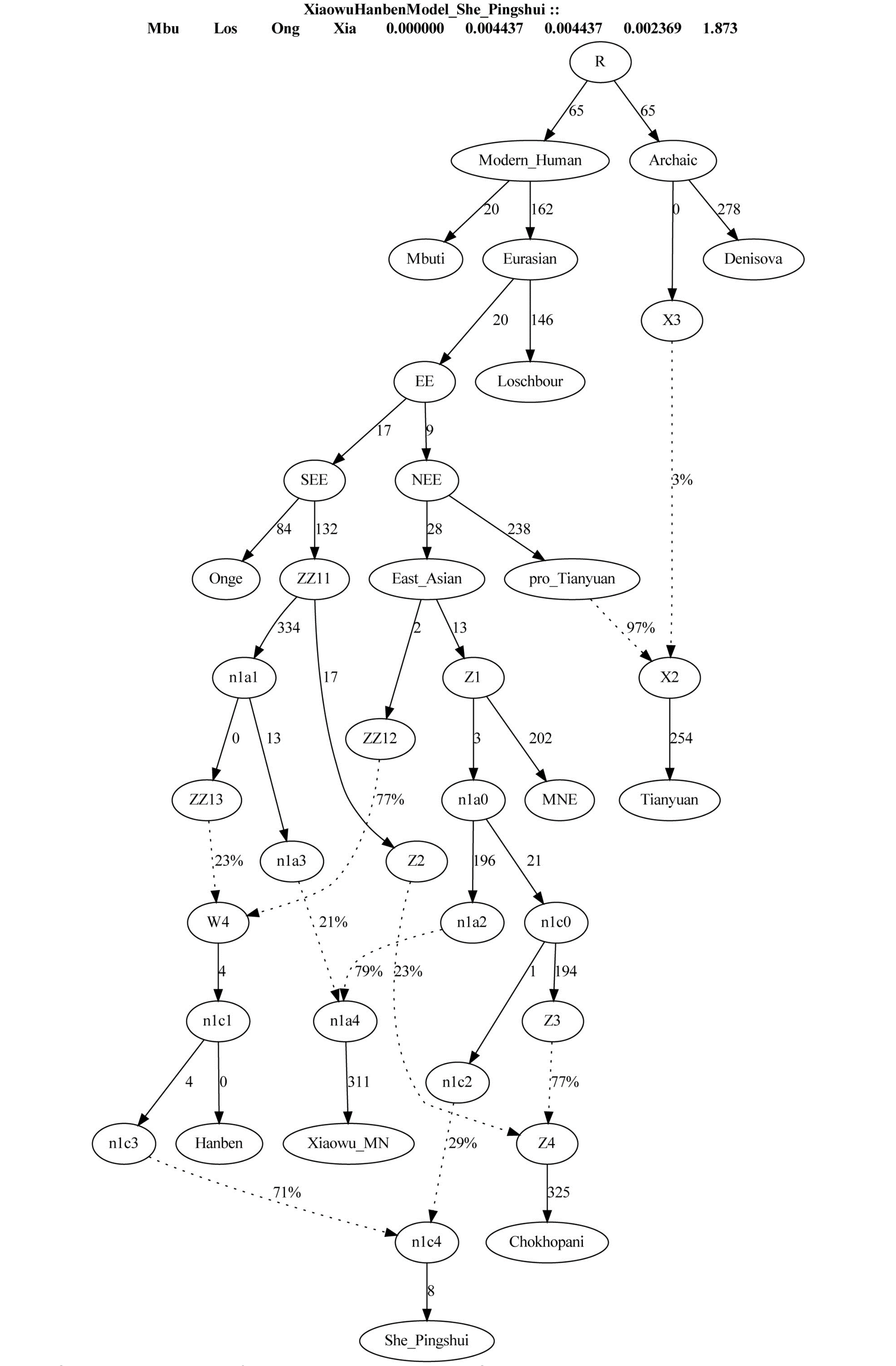

Figure S17. Demographic history of newly genotyped coastal Pingshui She population.
The presented qpGraph-based phylogenetic topology was fitted for Pingshui She people with the best-worst Z-score of 1.873. Branch lengths were labeled in units of f_2 genetic drift distance times 1000. The dotted line indicates the admixture events with admixture proportion.

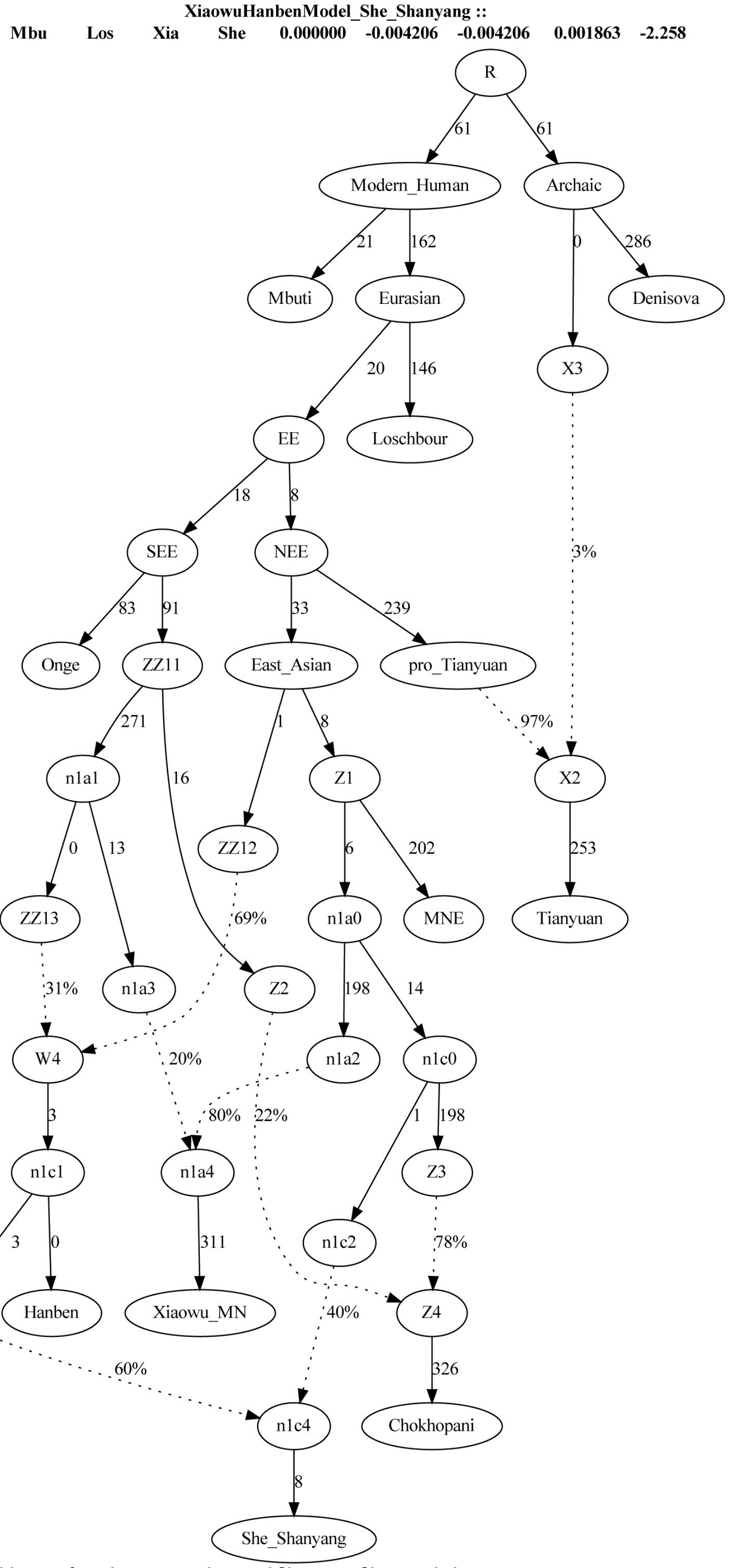

Figure S18. Demographic history of newly genotyped coastal Shanyang She population.
The presented qpGraph-based phylogenetic topology was fitted for Shanyang She people with the best-worst Z-score of -2.258. Branch lengths were labeled in units of f_2 genetic drift distance times 1000. The dotted line indicates the admixture events with admixture proportion.

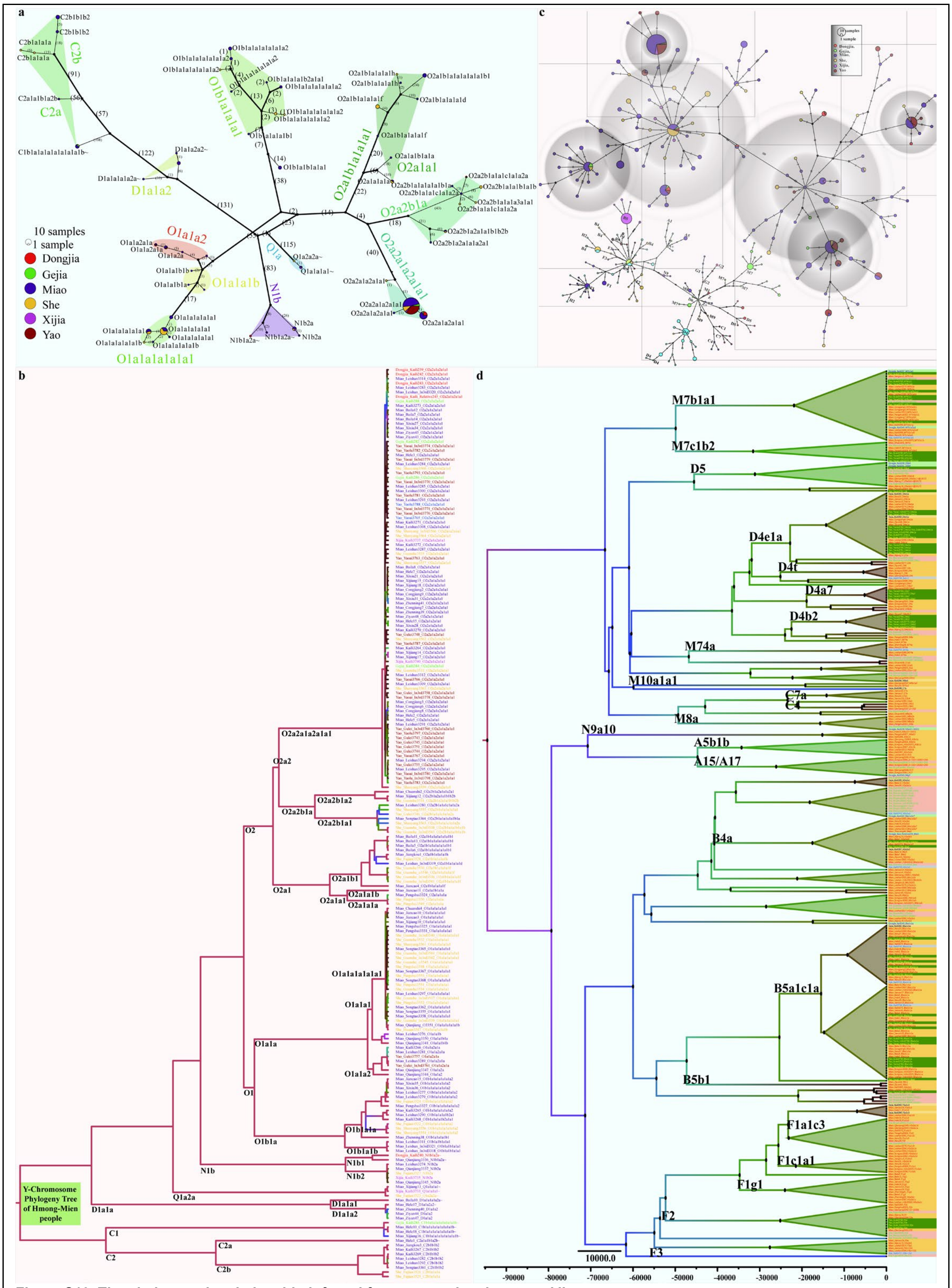

Figure S19. The phylogenetic relationship inferred from paternal and maternal lineages.
(**a**) Median-joining Network that was reconstructed via popART showed the distribution of paternal lineages. (**b**) The clustering patterns of individual lineage inferred from the Y-LineageTracker. (**c**) The Network-based phylogenetic relationship showed the maternal genetic history of HM people. Some star-like structures were emphasized using the gray color. The haplogroup distribution was shown in the left bottom network topology. (**d**) The constructed mtDNA phylogeny showed the patterns of population divergence, expansion and admixture among geographically different HM people.

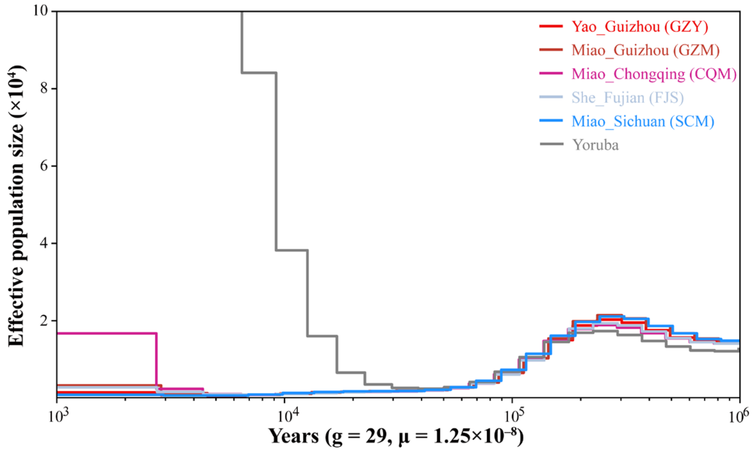

Figure S20. The effective population sizes (Ne) of inland and coastal HM-speaking populations, and the Yoruba genome was added as a benchmark. The Ne of geographically diverse HM-speaking populations was zoomed in Figure 3a.

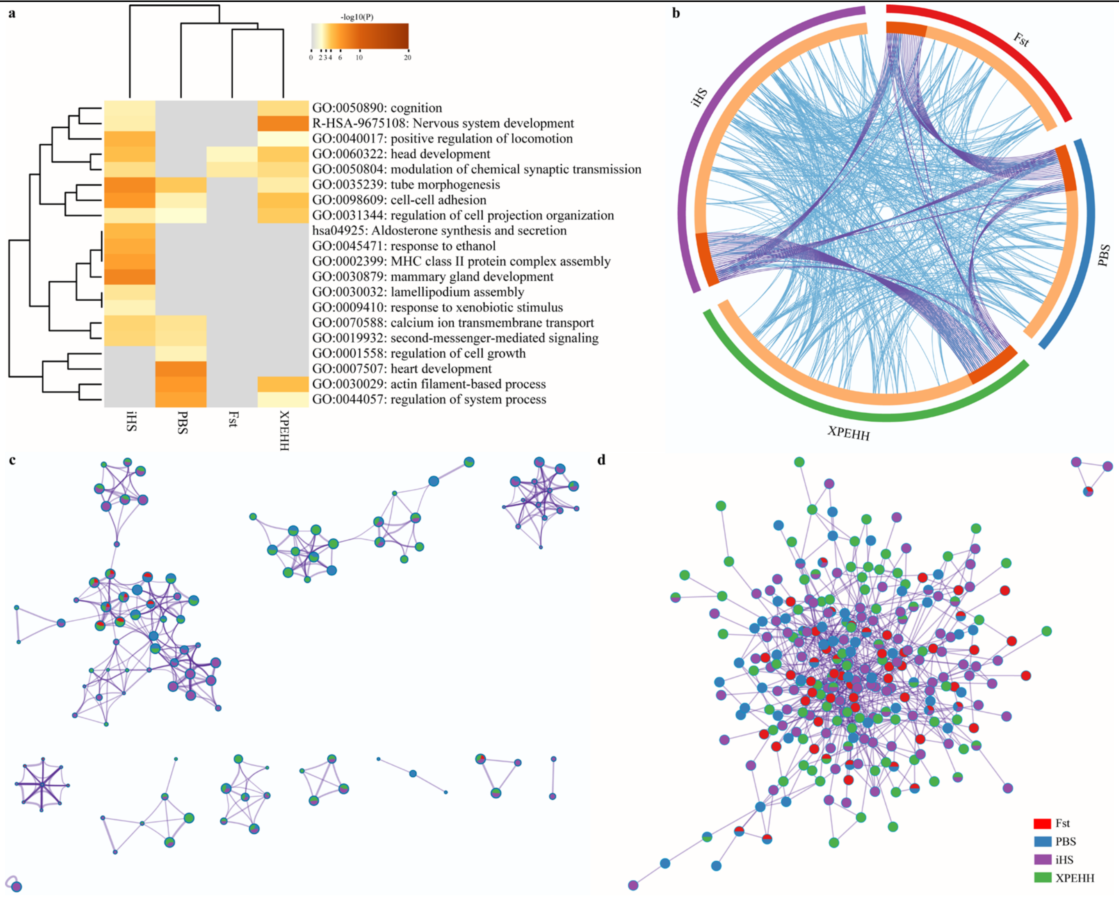

Figure S21. The annotation results of candidate signatures of natural selection identified based on PBS, Fst, iHS, and XPEHH approaches.

(**a**) Heatmap of enriched biological pathways across input gene lists, colored by p-value. (**b**) The Circos plot showed the overlap of candidate genes identified between different approaches. Each arc on the outside represents the identity of each gene list. Each gene member in the corresponding list is assigned a point on the inside arc. The dark orange color represents the genes that are shared by multiple lists and the light orange color represents genes that are unique to one gene list. Purple lines link the same gene shared by multiple gene lists. Blue lines link the genes, although different, fall under the same ontology term. (**c**) The enriched ontology clusters were pied by gene counts across different approaches. Each pie sector is proportional to the number of hits originating from a gene list. The color code for the pie sector represents a gene list generated by each method. (**d**) The protein-protein interaction (PPI) network for merged gene lists. All input gene lists were merged into one list, resulting in a PPI network.

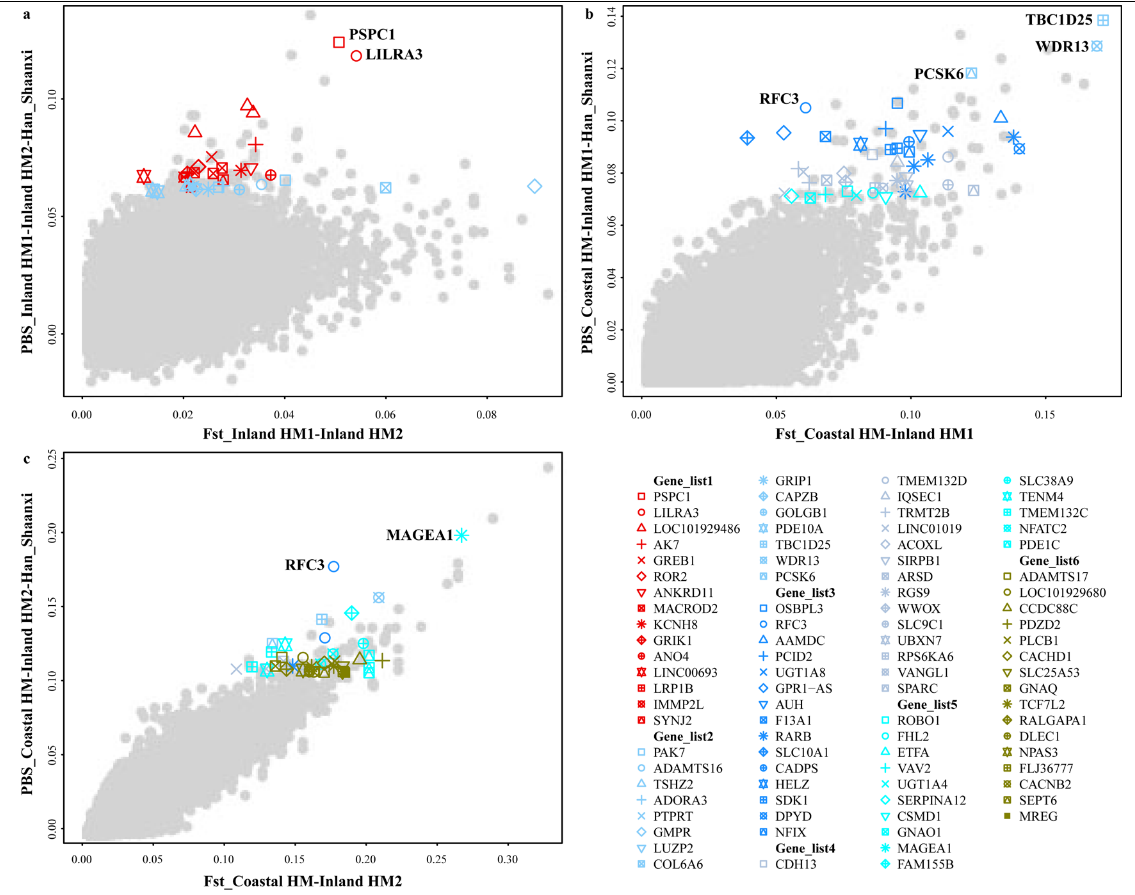

Figure S22. The signatures of natural selection specific to Inland HM1 and Coastal HM were identified based on the PBS approach.
(**a**) The candidate genes of natural selection identified in Inland HM1 relative to Inland HM2. The X-axis denotes the Fst values for each SNP between Inland HM1 and Inland HM2. (**b**) The signatures of natural selection identified in Coastal HM based on the PBS values of Coastal HM-Inland HM1-Han_Shaanxi trio model. The X-axis suggests the Fst values for each SNP between Coastal HM and Inland HM1. (**c**) The natural selection signals identified in Coastal HM relative to Inland HM2. The X-axis denotes the Fst values for each SNP between Coastal HM and Inland HM2. The top 100 candidate genes located in the coding regions were highlighted.

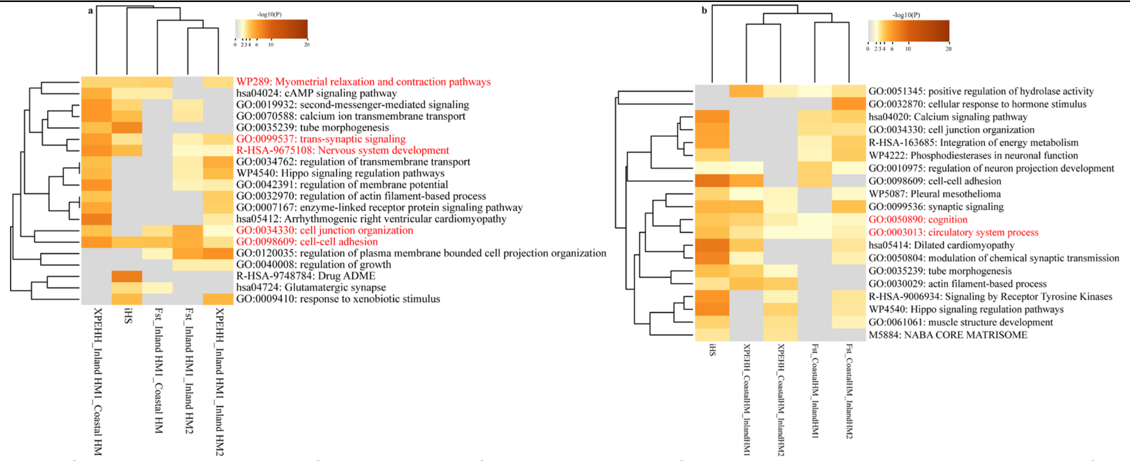

Figure S23. The annotation results of candidate genes of natural selection specific to one regional HM-speaking population identified based on Fst, iHS, and XPEHH approaches.
(**a**) Heatmap of enriched biological pathways across input gene lists identified in Inland HM1, colored by p-value. (**b**) Heatmap of enriched biological pathways across input gene lists identified in Coastal HM, colored by p-value.
